# Globally elevated chemical weathering rates beneath glaciers

**DOI:** 10.1038/s41467-022-28032-1

**Published:** 2022-01-20

**Authors:** Xiangying Li, Ninglian Wang, Yongjian Ding, Jon R. Hawkings, Jacob C. Yde, Robert Raiswell, Jintao Liu, Shiqiang Zhang, Shichang Kang, Rongjun Wang, Qiao Liu, Shiyin Liu, Roland Bol, Xiaoni You, Guoyu Li

**Affiliations:** 1 Shaanxi Key Laboratory of Earth Surface System and Environmental Carrying Capacity, Northwest University, Xi’an, China; 2 College of Urban and Environmental Sciences, Northwest University, Xi’an, China; 3 State Key Laboratory of Frozen Soil Engineering, Northwest Institute of Eco-Environment and Resources, Chinese Academy of Sciences, Lanzhou, China; 4 CAS Center for Excellence in Tibetan Plateau Earth Sciences, Beijing, China; 5 State Key Laboratory of Cryospheric Science, Northwest Institute of Eco-Environment and Resources, Chinese Academy of Sciences, Lanzhou, China; 6 University of Chinese Academy of Sciences, Beijing, China; 7 China-Pakistan Joint Research Center on Earth Sciences, CAS-HEC, Islamabad, Pakistan; 8 Department of Earth and Environmental Science, University of Pennsylvania, Philadelphia, PA USA; 9 Department of Environmental Sciences, Western Norway University of Applied Sciences, Sogndal, Norway; 10 Cohen Biogeochemistry Laboratory, School of Earth and Environment, University of Leeds, Leeds, UK; 11 College of Hydrology and Water Resources, Hohai University, Nanjing, China; 12 Institute of Mountain Hazards and Environment, Chinese Academy of Sciences, Chengdu, China; 13 Institute of International Rivers and Eco-security, Yunnan University, Kunming, China; 14 Forschungszentrum Jülich IBG-3, Wilhelm-Johnen-Straße, Jülich, Germany; 15 School of Natural Sciences, Environment Centre Wales, Bangor University, Bangor, UK; 16 College of Resources and Environmental Engineering, Tianshui Normal University, Tianshui, China

**Keywords:** Cryospheric science, Hydrology

## Abstract

Physical erosion and chemical weathering rates beneath glaciers are expected to increase in a warming climate with enhanced melting but are poorly constrained. We present a global dataset of cations in meltwaters of 77 glaciers, including new data from 19 Asian glaciers. Our study shows that contemporary cation denudation rates (CDRs) beneath glaciers (2174 ± 977 Σ*meq^+^ m^−^^2^ year^−^^1^) are ~3 times higher than two decades ago, up to 10 times higher than ice sheet catchments (~150-2000 Σ*meq^+^ m^−^^2^ year^−^^1^), up to 50 times higher than whole ice sheet means (~30-45 Σ*meq^+^ m^−^^2^ year^−^^1^) and ~4 times higher than major non-glacial riverine means (~500 Σ*meq^+^ m^−2^ year^−^^1^). Glacial CDRs are positively correlated with air temperature, suggesting glacial chemical weathering yields are likely to increase in future. Our findings highlight that chemical weathering beneath glaciers is more intense than many other terrestrial systems and may become increasingly important for regional biogeochemical cycles.

## Abstract (web summary)

Global glacial chemical denudation is one of the largest contributors to global elemental cycles and, amplified by climate warming, will significantly impact nutrient loads in downstream ecosystems.

## Introduction

Glaciers (here defined to include all glaciers but exclude ice sheets) and ice sheets play a substantial role in the global cycles of water^1–3^, sediment^4–6^, and elements^7–12^. The highly efficient physical erosion beneath glaciers creates an abundance of reactive mineral surfaces^13,14^, and the resultant glacier flour is susceptible to rapid chemical weathering because of very high microparticle surface areas^15^ and amorphous mineral coatings^16–18^. Chemical weathering associated with glaciers (and ice sheets) may also be a CO_2_ sink or source via dissolution of bedrock minerals (e.g., silicate, carbonate, and sulfide), thereby influencing carbon cycles and global climate^16,19–23^.

Chemical weathering rates can be estimated using cation yields derived from mineral weathering reactions (referred to as crustal cation denudation rate or CDR)^16,21,24–27^ because major cations (K^+^, Na^+^, Mg^2+^, and Ca^2+^) in meltwaters are predominantly derived from crustal mineral weathering (with the proportion of ~70–99% being of total four cations)^21,24,27–32^. To date, glacial CDRs have been quantified at almost thirty basins with published values ranging from ~36 to 56 Σ*meq^+^ m^−^^2^ year^−^^1^ (Watson River in Greenland^33^) to ~4160 Σ*meq^+^ m^−^^2^ year^−^^1^ (Dokriani Glacier in the Himalayas^34^). Global mean CDR was estimated at 740 ± 830 Σ*meq^+^ m^−^^2^ year^−^^1^ for glaciers before 2000 (calculated from published CDRs at glacial basins in Asia, Alaska, Arctic Canada, Europe, Iceland, Svalbard, and Western Canada and USA sampled during 1963–1999)^16,21,24,27,28,34–40^, which is almost two times higher than global continental mean CDR (~380–390 Σ*meq^+^ m^−^^2^ year^−^^1^)^16,41–43^. However, previous estimates of CDRs in glacial catchments are outdated and incomplete^16,44–46^. This study will update these estimates and identify the chemical and physical sampling needed for future progress.

Here we present concentrations of major cations in meltwaters of the 19 glaciers within Asia. The new data is combined with a global analysis of CDRs in glacial environments to help elucidate the role of future climate-driven glacier retreat and meltwater export in regional chemical weathering elemental cycles. We propose that the subglacial chemical weathering rates are directly correlated to changes in climate and glacial runoff, and subglacial chemical weathering will become increasingly important for regional elemental cycles and weathering-climate feedbacks in response to accelerated global glacier (and ice sheet) mass loss in a warming environment^47–51^.

## Results and discussion

### Glacial cation concentrations

**Fig. 1:**
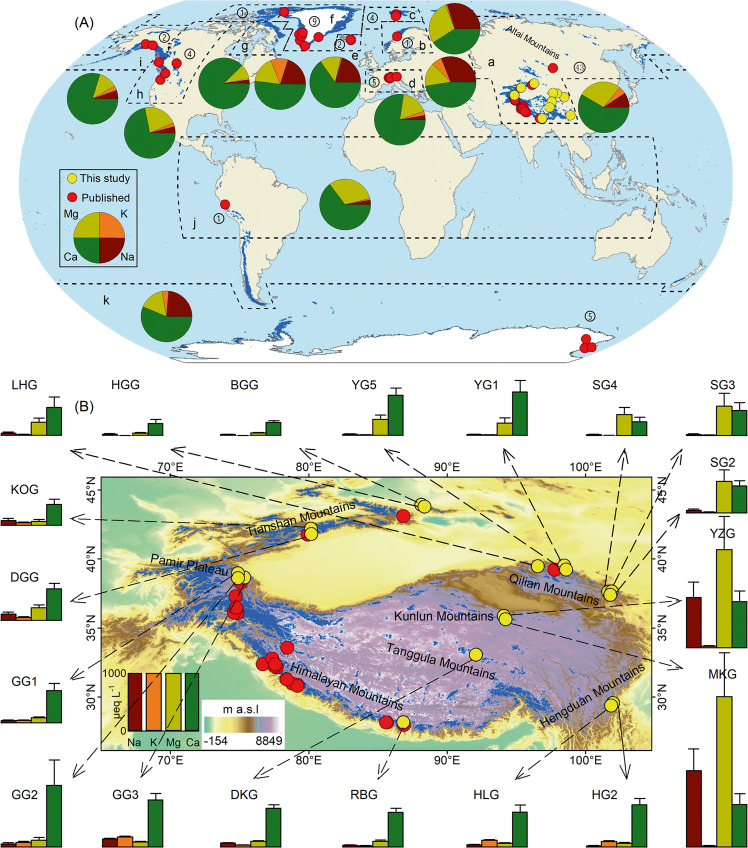
Location of the 77 glaciers (red and yellow dots) and the percentage of mean cation concentration from eleven glacial regions (pie) globally. **a** 43 glaciers in eight mountain ranges within Asia, including the Altai (ATG; **A**), the Tianshan (TSG), the Qilian (QLG), the Kunlun (KLG), the Tanggula (TGG), the Pamir (PAG), the Hengduan (HDG), and the Himalayan (HMG) mountain ranges (**B**). Note that the mean cation concentrations from the 19 glaciers (yellow dots and bar plots; the horizontal lines above the bar plots indicate the standard deviations) in the Asian glacial region (ASG) were sampled as part of this study (**B**; Supplementary Tables 1–4). **b**–**k** 34 glaciers in other ten glacial regions outside of Asia (**A**; Supplementary Tables 1, 3 and 5), including Scandinavia (SCG; **b**), Svalbard and Jan Mayen (SJG; **c**), Central Europe (CEG; **d**), Iceland (ICG; **e**), Greenland Periphery (GPG; **f**), Arctic Canada (ACG; **g**), Western Canada and USA (CUG; **h**), Alaska (ALG; **i**), low Latitudes (LLG; **j**), and Antarctic and Subantarctic (ANG; **k**). Note that glaciers are shown in blue, and the numbers of glaciers used in this study are marked in each glacial region.

**Fig. 2:**
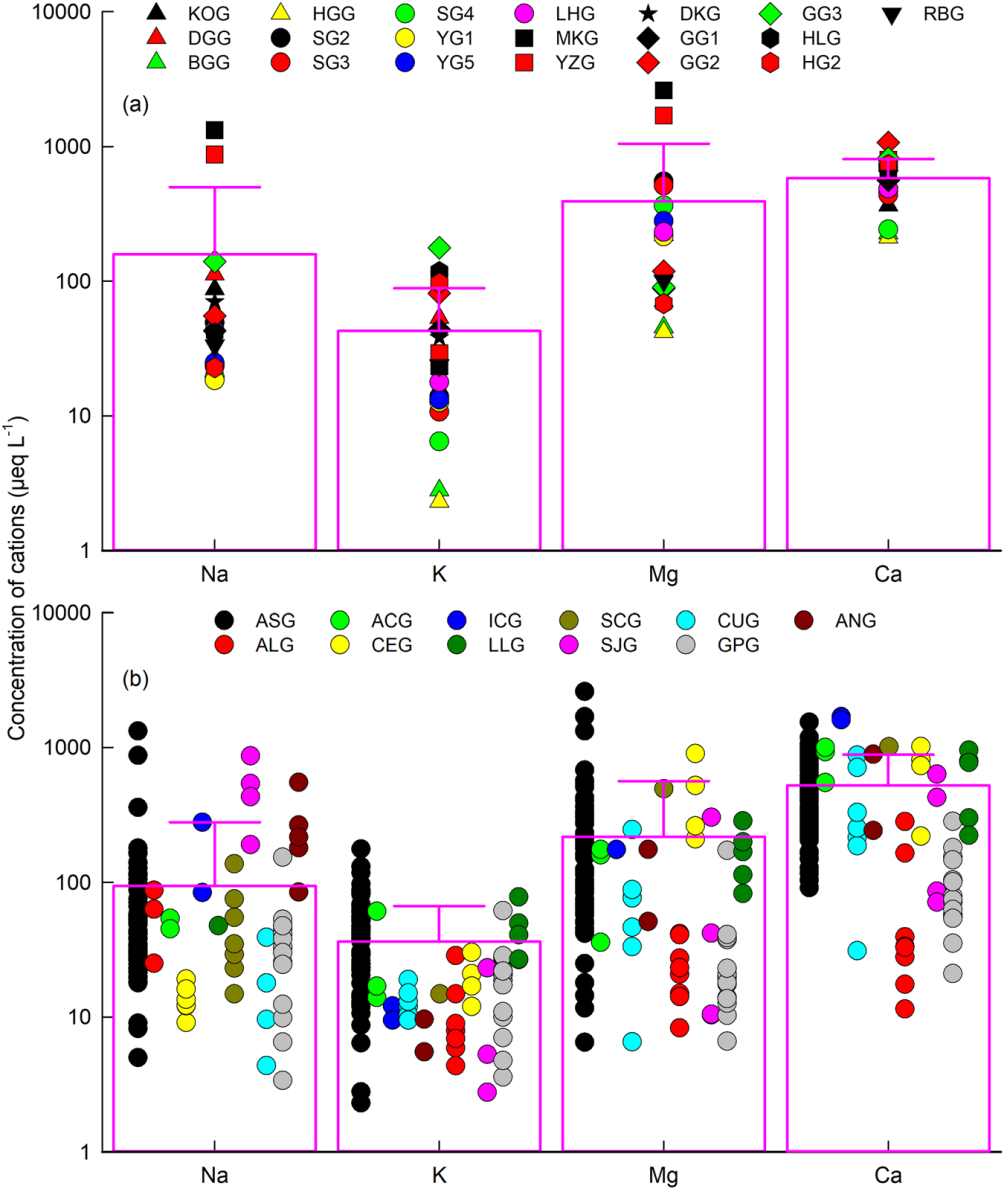
Mean cation concentrations in meltwaters for glaciers globally. **a** 19 glaciers in Asia generated in this study (Supplementary Table 4); **b** 77 glaciers in eleven glacial regions (Supplementary Tables 3 and 5). The boxes and horizon lines above indicate the mean cation concentration and standard deviations, respectively.

We developed a global database of major cation concentrations from 77 glaciers (63 mountain/valley glaciers and 14 ice sheet outlet glaciers) containing 5465 samples generated in this study and published literature (Fig. 1A and Supplementary Tables 1–3). For our 19 Asian glaciers, the dominant cation is Ca^2+^ for 14 of the 19 glaciers, followed by Mg^2+^, Na^+^, and K^+^ (Fig. 1B). This is consistent with published cation data from glaciers in Asia (Supplementary Fig. 1). However, at SG2, SG3, and SG4 in the Qilian and at MKG and YZG in the Kunlun, meltwater compositions are dominated by Mg^2+^ (Fig. 1B), suggesting the contribution of magnesium-rich minerals^52–54^. Globally, Ca^2+^ is also the dominant cation in most glacial basins (Supplementary Fig. 1b). However, the dominant cation is Mg^2+^ in some basins within Asia, and Na^+^ and K^+^ in Scandinavia and Svalbard. The basin-mean cation concentrations range between one and two orders of magnitude, with arithmetic mean of 159 ± 341 μeq L^−^^1^ for Na^+^, 42.7 ± 46.0 μeq L^−^^1^ for K^+^, 393 ± 655 μeq L^−^^1^ for Mg^2+^, and 581 ± 225 μeq L^−^^1^ for Ca^2+^ for the 19 Asian glaciers (Fig. 2a and Supplementary Table 4).

The range of cation concentrations for the 19 Asian glaciers (where data from 4 glaciers extended existing records) overlaps the majority of previously published data from 28 glaciers in Asia and from other glaciers worldwide (Supplementary Fig. 2). However, mean cation concentrations for the 19 Asian glaciers are generally higher than previously published cation means for Asian glaciers and worldwide glaciers. For the 77 glaciers in the current global data set mean cation concentrations vary over two to three orders of magnitude (3.38 to 1323 μeq L^−^^1^ for Na^+^, 2.31 to 177 μeq L^−^^1^ for K^+^, 6.50 to 2605 μeq L^−^^1^ for Mg^2+^, and 11.5 to 1701 μeq L^−^^1^ for Ca^2+^; Fig. 2b). Mean concentrations from glaciers (93.3 ± 177 μeq L^−^^1^ for Na^+^, 34.6 ± 29.0 μeq L^−^^1^ for K^+^, 191 ± 321 μeq L^−^^1^ for Mg^2+^, and 472 ± 366 μeq L^−^^1^ for Ca^2+^; Supplementary Table 5) are comparable to those from global non-glacial rivers (265 μeq L^−^^1^ for Na^+^, 59.0 μeq L^−^^1^ for K^+^, 342 μeq L^−^^1^ for Mg^2+^, and 900 μeq L^−^^1^ for Ca^2+^) after accounting for the uncertainty^55,56^.

### Diurnal variations in cation concentration

**Fig. 3:**
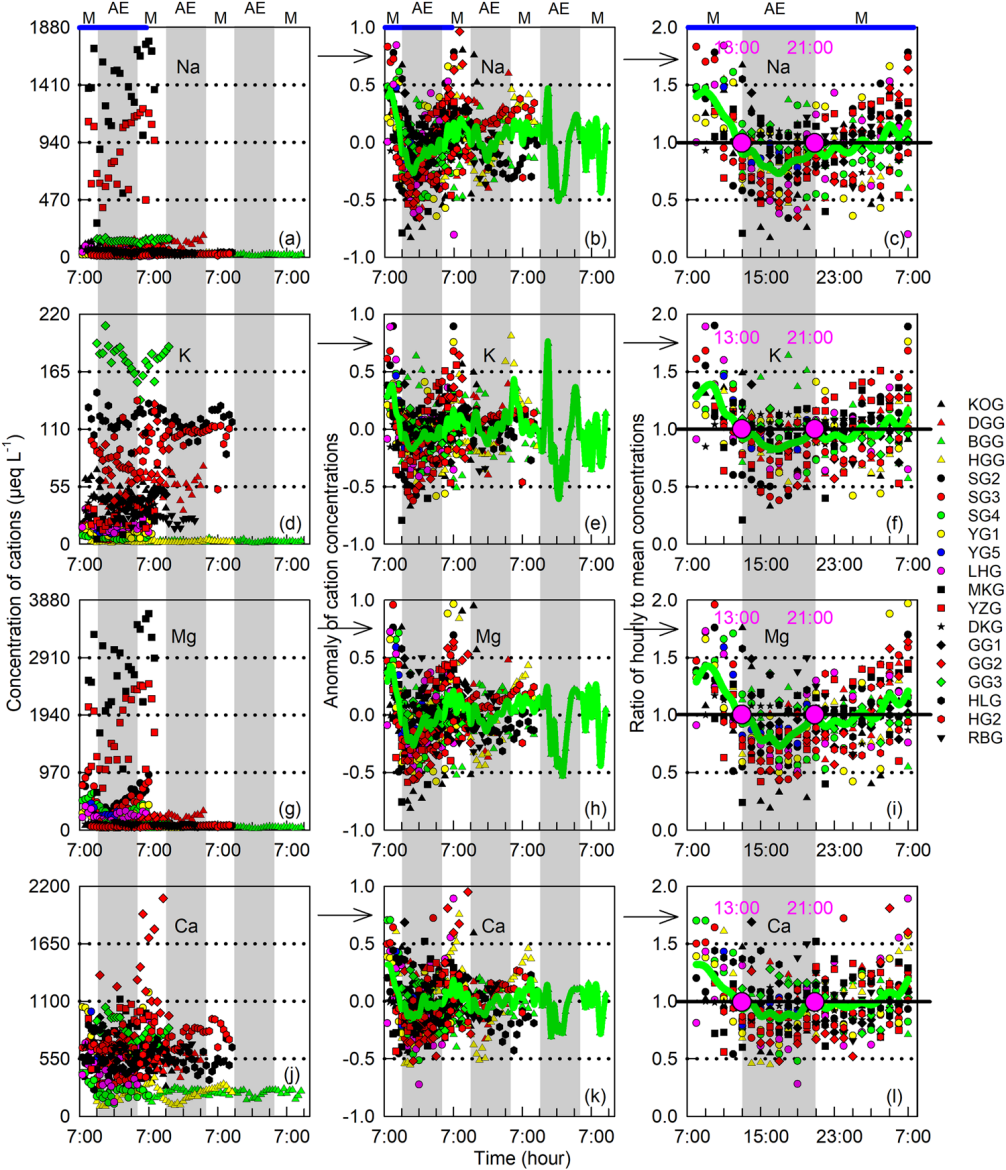
Hourly variations in cation concentration as well as the anomalies and ratios for the 19 Asian glaciers generated in this study over a three-day period. **a**, **d**, **g**, **j** Hourly concentrations; **b**, **e**, **h**, **k** Anomalies of hourly concentration (i.e., the difference between hourly and mean concentrations divided by mean concentrations); **c**, **f**, **i**, **l** Ratios of hourly to mean concentrations. Note that the diurnal changes were indicated by gray shadows (M and AE indicate the morning and afternoon/evening, respectively), the mean anomalies and ratios were indicated by green solid lines, and the pink dots indicate the time at 13:00 and 21:00 when hourly concentrations equal to the daily mean.

For the 19 Asian glaciers, hourly cation concentrations differ between one to three orders of magnitude (Fig. 3a, d, g, j) and display strong diurnal trends, with higher values in the morning hours (22:00–12:00 h the following day) and lower values in the afternoon and evening hours (13:00–21:00 h; Fig. 3b, e, h, k). Compared with seasonal change of glacial cations^30,45,46,57–59^, the diurnal changes reflect the interaction time between water and rock/sediment coupled with meltwater dilution^26,60,61^. Field observation showed that during the morning hours lower air temperature reduces the supraglacial melt, leading to less meltwater, lower proglacial runoff, and higher cation concentrations^26,60–62^. Conversely, cation concentrations are diluted by increasing meltwater discharge and reduced transit times in the subglacial hydrological system later in the day (Fig. 3b, e, h, k). Our data are consistent with the diurnal changes for glacial runoff cations and/or electrical conductivity from Haut Glacier d’Arolla in Central Europe^61^, and from Qiyi Glacier and Dongkemadi Glacier in Asia^26,60,62^. To identify the “most representative” daily sampling time, the ratios of hourly to daily mean cation concentrations were calculated (Fig. 3c, f, i, l). The hourly ratios range from 0.88 to 1.44 (with an average of 1.11) for Na^+^, 0.91 to 1.37 (1.08) for K^+^, 0.90 to 1.42 (1.11) for Mg^2+^, and 0.94 to 1.32 (1.07) for Ca^2+^ during the morning hours with high cation concentration and low runoff, in comparison to from 0.73 to 0.94 (0.85) for Na^+^, 0.83 to 0.98 (0.90) for K^+^, 0.73 to 0.98 (0.86) for Mg^2+^, and 0.85 to 1.04 (0.93) for Ca^2+^ during the afternoon and evening hours with low cation concentration and high runoff. The daily amplitude of these ratios provides a template that sampling at ~13:00 h or ~21:00 h can capture the “most representative” meltwater (Fig. 3c, f, i, l). This can be used to help estimate the daily mean concentrations and therefore the fluxes of glacial cations in future work on similar glacial systems.

### Glacial chemical weathering and cation sources

**Fig. 4:**
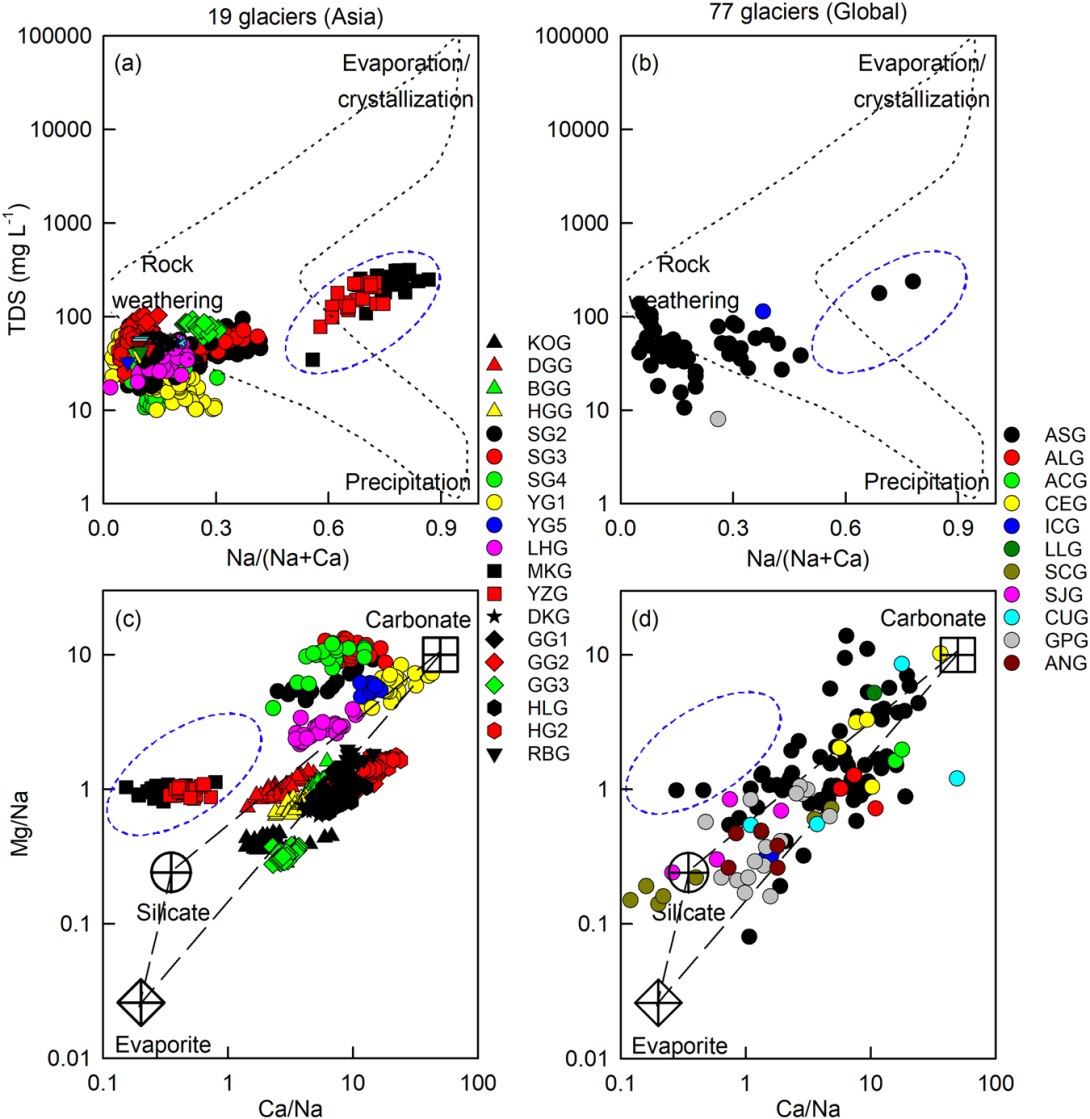
Chemical weathering and cation sources for glaciers globally. **a**, **b** Gibbs plots [total dissolved solids (TDS) versus molar Na/(Na+Ca)], and **c**, **d** Mixing diagrams of Na-normalized molar ratios for the 19 Asian glaciers generated in this study and for 77 glaciers in eleven glacial regions (Supplementary Table 6). Note that data within the blue dashed circles denote Meikuang Glacier (MKG) and Yuzhufeng Glacier (YZG) in the Kunlun (Fig. 1B), and cations are not corrected for the sea-salt and aerosol contributions.

Glacier meltwater chemistry is related to the principle weathering reactions, rock mineralogy, and drainage system configuration^19,21,41,57,63^. The Gibbs plots and elemental stoichiometry were used to identify the sources of cations^13,63,64^. Cation weathering products from glacial basins and regions confirm the dominance of rock weathering as the major source (Fig. 4a, b). Notably samples from MKG and YZG plot away from the other glaciers, which may be related to differences in the underlying geology. Na-normalized ratios for Ca^2+^ and Mg^2+^ are a useful tool for exploring the relative importance of different weathering processes^26,33,65^. Clusters from the 19 Asian glaciers are widely distributed between silicate and carbonate end-members, but MKG and YZG are close to the silicate end-member (Fig. 4c). This suggests that glacial chemical weathering in the Kunlun has a higher relative proportion of silicate to carbonate weathering than in other mountain ranges within Asia. In detail, carbonate weathering dominates the weathering regime for glaciers in Asia, Low Latitudes, Arctic Canada and Europe; however, in Svalbard, Iceland, Scandinavia, Greenland, and Antarctic and Subantarctic silicate weathering appears to be comparatively more important (Fig. 4d and Supplementary Table 6).

The chemical composition of meltwaters has been shown to be typically controlled by the presence of carbonate weathering in most glacial environments^6,13,26,27,32,34,41,45,46,60,66^. Carbonate minerals (e.g., calcite, dolomite, and siderite) are preferentially weathered from glacial flour even if bulk bedrock is dominated by silicate minerals (e.g., potash feldspar, plagioclase, and illite)^13^. Previous studies also indicate that Ca^2+^ and Mg^2+^ are primarily derived from carbonate weathering and Na^+^ and K^+^ from silicate weathering in glacial environments^19,27,67–69^. Our data set is consistent with this, reflected by higher contributions of Ca^2+^ and Mg^2+^ to total cation load from the 19 Asian glaciers (71–97%) and from previously published glaciers (51–98%; except for Scott Turnerbreen with 49%; Supplementary Tables 3–5)^31^. In detail, dolomite may be a source of Ca^2+^ and Mg^2+^ at SG2, SG3, and SG4 which is supported by their similar concentrations (Supplementary Table 3 and Table 7). However, at MKG and YZG silicate and evaporite may be a key source of Mg^2+^ and Na^+^ which fits with higher concentrations of Mg^2+^ and Na^+^ than Ca^2+^ in glacial meltwaters (Fig. 4c, d).

### Export of cations from glaciers

Estimates of cation fluxes should ideally be based on the discharge-weighted or mid-summer normalized cation data (to correct for variable flow and associated concentration effects; Figs. 2 and 3)^26,45,46,57,61^. However, our global compilation of glacial cations (Supplementary Table 3) shows that discharge data are sparse and available for only a few glaciers. Future studies need to address this omission which will seriously hamper attempts to compare the regional weathering rates and generate accurate flux (and CDRs) estimates. Here we proceed to use our mean cation concentration data in combination with modeled meltwater flux data to produce a first-order estimate of regional cation fluxes from glaciers in Asia and other glacial regions and then generate a global estimate (Table 1). This approach is tested against estimates based on the methods of regional discharge-weighted cation concentration and regional discharge extrapolation using all data in nine non-ice sheet glacial regions (apart from Greenland Periphery, and Antarctic and Subantarctic) and the mid-summer data in six non-ice sheet glacial regions (apart from Alaska, Arctic Canada, and Svalbard), respectively, in the current global data set (Fig. 1A and Supplementary Table 8).

**Table 1:** Cation flux (±std; Gg year^−^^1^) and crustal-derived cation denudation rate (CDR; ± std; Σ*meq^+^ m^−^^2^ year^−^^1^) for glaciers in eight mountain ranges within Asia, for glaciers in ten glacial regions (excluding ANG) globally, and for glaciers and ice sheets over the world, in comparison to the latitude (LAT; °), glacial area (ARE; km^2^), glacial runoff (RUN; km^3^ year^−^^1^), specific discharge (SQ; m year^−^^1^), mean annual air temperature (MAT; °C), and mean annual precipitation (MAP; mm).

	LAT^a^	ARE	RUN	SQ^b^	MAT^c^	MAP^c^	Cation flux					CDR^d^	*N*
							Na^+^	K^+^	Mg^2+^	Ca^2+^	Total		
Ranges													
ATG	48	179^e^	0.65^f^	3.657	−2.95	516	0.30 ± N/A	0.52 ± N/A	1.20 ± N/A	9.82 ± N/A	11.8 ± N/A	3425 ± N/A	1
TSG	42	7180^e^	8.44^f^	1.175	0.77	687	17.1 ± 19.0	14.1 ± 11.5	16.0 ± 13.8	109 ± 47.5	156 ± 91.8	1076 ± 622	942
QLG	38	1598^e^	1.22^f^	0.761	−2.26	486	1.42 ± 1.06	0.66 ± 0.21	6.06 ± 2.44	16.9 ± 9.46	25.0 ± 13.2	881 ± 448	260
KLG	37	11,524^e^	5.00^f^	0.434	-6.43	412	126 ± 36.6	5.12 ± 0.82	129 ± 38.3	76.9 ± 4.60	337 ± 80.3	1646 ± 405	48
TGG	33	1844^e^	1.16^f^	0.629	−6.56	753	1.44 ± 0.62	1.15 ± 0.81	1.30 ± 0.18	14.4 ± 1.74	18.3 ± 3.35	491 ± 78.2	71
PAG	39	2160^e^	4.68^f^	2.169	−2.42	877	6.65 ± 6.04	14.2 ± 13.2	4.39 ± 2.51	64.6 ± 30.6	89.8 ± 52.4	1936 ± 1057	88
HDG	29	1395^e^	0.90^f^	0.646	2.24	1650	0.59 ± 0.21	3.21 ± 0.76	0.67 ± 0.09	10.4 ± 2.25	14.9 ± 3.31	486 ± 105	623
HMG	31	6821^e^	19.4^f^	2.841	4.89	1889	24.8 ± 18.7	36.2 ± 15.3	43.0 ± 50.7	167 ± 112	271 ± 197	2000 ± 1586	1528
Regions													
ASG	52	121,694^g^	359^h^	2.950	−2.66	550	723 ± 1528	634 ± 440	1074 ± 1680	3873 ± 2195	6305 ± 5843	2655 ± 2565	3561
ALG	61	86,715^g^	338^h^	3.898	−4.68	800	453 ± 243	404 ± 347	503 ± 311	5597 ± 1645	6958 ± 2545	3925 ± 1399	158
ACG	71	145,767^g^	212^h^	1.454	−14.1	353	241 ± 31.0	89.3 ± 15.0	446 ± 1.80	7019 ± 273	7796 ± 321	2696 ± 103	46
CEG	45	2063^g^	9^h^	4.363	10.2	948	2.83 ± 0.72	4.63 ± 1.22	8.88 ± 8.42	67.0 ± 55.2	83.4 ± 65.5	2088 ± 1701	363
ICG	63	11,060^g^	51^h^	4.611	0.12	1416	212 ± 161	15.1 ± 5.78	69.5 ± 54.0	579 ± 468	876 ± 689	3682 ± 2918	19
LLG	3	2346^g^	15^h^	6.394	24.4	1378	16.4 ± N/A	8.72 ± N/A	89.1 ± N/A	305 ± N/A	419 ± N/A	9683 ± N/A	16
SCG	66	2851^g^	11^h^	3.858	2.18	866	12.6 ± 10.1	4.48 ± 3.45	3.16 ± 1.61	16.8 ± 21.3	37.0 ± 36.4	545 ± 547	N/A
SJG	77	33,922^g^	73^h^	2.152	−9.27	564	850 ± 471	57.1 ± 22.0	417 ± 278	1018 ± 496	2342 ± 1267	2699 ± 1483	454
CUG	55	14,559^g^	62^h^	4.259	−0.24	574	25.2 ± 21.8	25.2 ± 26.9	68.4 ± 106	379 ± 342	498 ± 497	1749 ± 1817	>24
GPG	72	89,721^g^	149^h^	1.661	−19.7	360	129 ± 118	118 ± 79.4	56.6 ± 70.4	290 ± 197	593 ± 465	285 ± 233	807
Glaciers													
Global	N/A	726,792^g^	1430^h^	1.968	N/A	N/A	3212 ± 3164	1573 ± 1104	3391 ± 3131	23,859 ± 7047	32,035 ± 14,446	2174 ± 977	>4641
Ice sheets													
GIS	N/A	1,711,279^i^	542^j^	0.317	N/A	N/A	470 ± 428	428 ± 289	206 ± 256	1053 ± 717	2158 ± 1691	54.4 ± 44.5	807
			652^k^	0.381	N/A	N/A	566 ± 515	515 ± 348	248 ± 308	1267 ± 863	2595 ± 2034	65.5 ± 53.5	807
AIS	N/A	12,295,000^l^	55^m^	0.004	N/A	N/A	328 ± 223	95.5 ± 45.6	112 ± 52.4	673 ± 360	1209 ± 681	4.51 ± 2.49	17
			254^m^	0.021	N/A	N/A	1515 ± 1028	441 ± 211	519 ± 242	3109 ± 1664	5584 ± 3144	20.8 ± 11.5	17
Global	N/A	14,006,279^i, l^	597^j, m^	0.321	N/A	N/A	798 ± 651	524 ± 335	318 ± 308	1726 ± 1078	3367 ± 2371	29.5 ± 23.5	824
			906^k, m^	0.402	N/A	N/A	2080 ± 1543	956 ± 558	766 ± 550	4376 ± 2527	8179 ± 5178	43.1 ± 32.5	824
Rivers													
Global	N/A	N/A	38,452^n^	N/A	N/A	N/A	234,557^o^	88,440^o^	157,653^o^	692,136^o^	1172,786	N/A	N/A

The eight mountain ranges within Asia include the Altai (ATG), the Tianshan (TSG), the Qilian (QLG), the Kunlun (KLG), the Tanggula (TGG), the Pamir (PAG), the Hengduan (HDG), and the Himalayan (HMG) mountain ranges. The ten glacial regions globally include Asia (ASG), Alaska (ALG), Arctic Canada (ACG), Central Europe (CEG), Iceland (ICG), Low Latitudes (LLG), Scandinavia (SCG), Svalbard, and Jan Mayen (SJG), Western Canada and USA (CUG), and Greenland Periphery (GPG). Note that GIS and AIS denote Greenland Ice Sheet and Antarctic Ice Sheet, respectively. *N* denotes sample size.

Note: Cation fluxes were not corrected for the sea-salt and aerosol contributions, and N/A denotes no available data.

^a^Value denotes the middle latitudes of the geographical range in which the mountain ranges or glacial regions are located.

^b^Value from glacial runoff divided by glacial area due to no available data for glacial drainage area.

^c^Value from the ERA-Interim (ERA-I) datasets, which can be downloaded from the European Centre for Medium-Range Weather Forecasts (ECMWF)^96^. The spatial resolution of the data is ~20 km × 20 km on 60 vertical levels. Data on ‘2 m temperature’ and ‘total precipitation’ were downloaded in.netcdf format for the period January 1981 to December 2019, which overlaps, at least in part, with the time period over which most of cation data were collected. These were loaded into ArcGIS 10.5 using the ‘Conversion Tools’ and ‘Spatial Analysis Tools’. The Raster Calculator was used to compute mean values of air temperature and precipitation during 1981–2019, and the climatic values were extracted for each mountain range, glacial region and glacial basin.

^d^Value was corrected by the mean percentages of crustal-derived *Na^+^, *K^+^, *Mg^2+^ and *Ca^2+^ fluxes to the Na^+^, K^+^, Mg^2+^ and Ca^2+^ fluxes, respectively, from 2 glaciers^27,32^ for Asian mountain ranges and ASG (79.9% for Na^+^, 99.7% for K^+^, 98.6% for Mg^2+^, and 99.9% for Ca^2+^), from >8 glaciers^24,28–30^ for SJG (33.9%, 85.1%, 79.0%, and 98.9%), from 1 glacier^21^ for CEG (87.0%, 99.6%, 98.8%, and 100%), and from 1 glacier^31^ for GIS and AIS (67.0%, 94.8%, 92.2%, and 99.6%), as well as from all glaciers (apart from Kuannersuit Glacier) mentioned above for ALG, ACG, ICG, LLG, SCG, CUG and global glaciers (67.0%, 94.8%, 92.2%, and 99.6%).

^e^Value from Liu et al.^83^.

^f^Value from Wang^82^.

^g^Value from Pfeffer et al.^97^.

^h^Value from Bliss et al.^70^.

^i^Value from Pfeffer et al.^97^ and Kargel et al.^92^.

^j^Value (i.e., mean annual runoff during 2000–2012) from Hawkings et al.^9^ and Tedesco et al.^75^.

^k^Value (i.e., mean annual runoff during 2006–2016) from Lenaerts et al.^74^.

^l^Value from Kargel et al.^98^.

^m^Value from the average melt estimates for surface runoff during 1991–2000^77^ and the theoretical model predictions for basal melt rate of 65 km^3^ a^−^^1^ with a standard deviation of ±50% for the minimum and maximum estimates^78^.

^n^Value from a literature with a global long-term mean annual runoff of 38,452 km^3^ year^−^^1^ ^99^.

^o^Value was calculated by mean concentrations (265 μeq L^−^^1^ for Na^+^, 59.0 μeq L^−^^1^ for K^+^, 342 μeq L^−^^1^ for Mg^2+^, and 900 μeq L^−^^1^ for Ca^2+^)^55,56^ multiplied by discharge^99^.

**Fig. 5:**
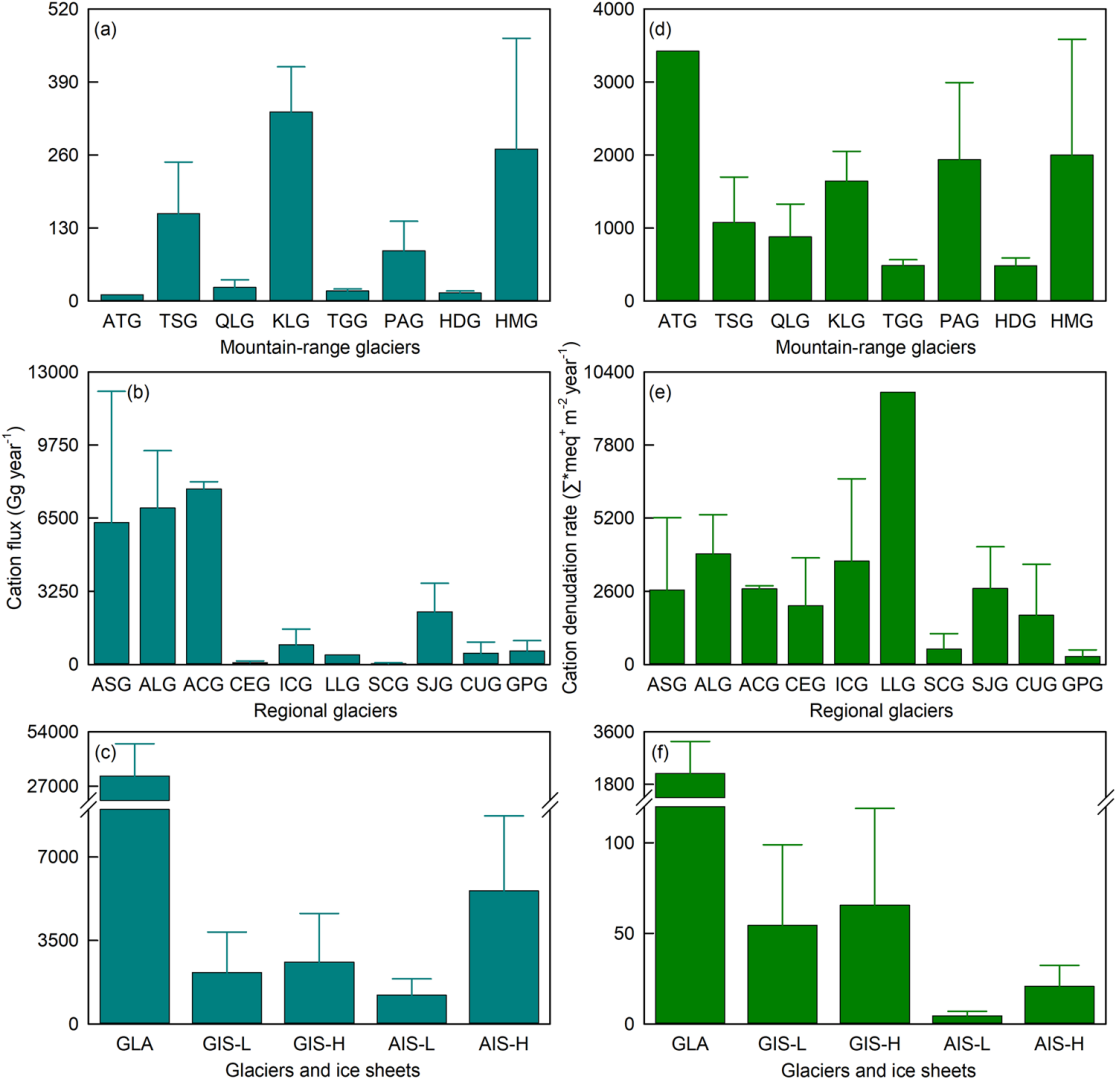
Cation flux and cation denudation rate for glaciers and ice sheets globally. **a**, **d** Glaciers in eight mountain ranges within Asia; **b**, **e** Glaciers in ten glacial regions (excluding ANG; Fig. 1); **c**, **f** Glaciers (GLA), Greenland Ice Sheet (GIS) and Antarctic Ice Sheet (AIS; Table 1). Note that L and H in the bottom plots (c, and f) denote the high- and low-end estimates, respectively, and the horizontal lines above the bars indicate the standard deviations.

Within Asia, the highest cation flux of 337 Gg year^−^^1^ is found in the Kunlun, which has the largest glacier cover (11,524 km^2^) and mean cation concentrations (apart from K^+^), despite the lowest specific discharge (0.43 m year^−^^1^) of mountain ranges (Fig. 5a and Table 1). The other two mountain ranges with large glacier cover, the Tianshan (7180 km^2^) and Himalayas (6821 km^2^), also have high cation fluxes of 156 Gg year^−^^1^ and 271 Gg year^−^^1^, respectively, compared to all other Asian glaciers. The smaller glacier-covered mountain ranges of Hengduan (1395 km^2^) and Altai (179 km^2^) have the lowest cation fluxes of 14.9 Gg year^−^^1^ and 11.8 Gg year^−^^1^, respectively (Fig. 5a). For the Asian glacial region (121,694 km^2^) we estimate a regional cation flux of 6305 ± 5843 Gg year^−^^1^ from Asian glaciers (Fig. 5b and Table 1). The glacial region with the highest glacier cover, Arctic Canada (145,767 km^2^), is also the region with the highest cation flux (7796 ± 321 Gg year^−^^1^). The lowest cation flux of 37.0 ± 36.4 Gg year^−^^1^ is found in Scandinavia, one of glacial regions with least glacier cover (2851 km^2^; Fig. 5b and Table 1).

For eight mountain ranges in Asia, glacial cation fluxes are linearly related to glacial area (*R*^2^ = 0.90, *p* < 0.01; Supplementary Fig. 3a). This indicates a relationship between glacial area and runoff for mountain ranges (*R*^2^ = 0.65, *p* < 0.02; Supplementary Fig. 3b). There are some exceptions to these trends. The Kunlun has a higher cation flux (337 Gg year^−^^1^) than the Himalayas (271 Gg year^−^^1^) even though glacial runoff is smaller (by around 5 km^3^ year^−^^1^; Table 1). For ten glacial regions (including Greenland Periphery), cation fluxes are also closely related to glacial area (*R*^2^ = 0.74, *p* < 0.01; Supplementary Fig. 3c), suggesting a close relationship between glacial area and melt (*R*^2^ = 0.95, *p* < 0.01; Supplementary Fig. 3d). Notably, cation export from Asia only accounts for 25% of that from nine non-ice sheet glacial regions (see above) despite its relatively large percentage (32%) of total runoff (Table 1). Conversely, glacial runoff from Arctic Canada accounts for 19% of total runoff yet appears to account for 31% of cation export. This indicates that using a single glacier or even a few glaciers to represent a whole glacial region may introduce large uncertainties into the calculations of cation export owing to large spatial variations of cation yield and/or weathering processes.

Given that glacial runoff from these nine non-ice sheet glacial regions (1130 km^3^ year^−^^1^; Table 1) accounts for 79.0% of total glacial runoff globally (excluding ice sheets)^70^, we can crudely estimate worldwide glacial cation export as 32,035 ± 14,446 Gg year^−^^1^ based on the discharge extrapolation (Table 1). This equals that calculated by the regional discharge-weighted mean cation concentrations from all glaciers in these nine non-ice sheet glacial regions multiplied by total glacial runoff (Supplementary Table 8). Based on mean cation concentrations from nine outlet glaciers in the Greenland Periphery^22,54,71–73^ (Supplementary Table 5) multiplied by total runoff (542–652 km^3^ year^−^^1^)^9,74,75^, total cation export from the Greenland Ice Sheet (GIS) was estimated as ranging from 2158 to 2595 Gg year^−^^1^ (Table 1), consistent with another recent estimate of cation release from the GIS (2386 Gg year^−^^1^)^76^. Cation release from the Antarctic Ice Sheet (AIS) was estimated as 1209 to 5584 Gg year^−^^1^ based on mean cation concentrations from five outlet glaciers in the Antarctic and Subantarctic multiplied by total runoff (55–254 km^3^ year^−^^1^)^77,78^ (Table 1 and Supplementary Table 5).

Cation export from glaciers wordwide is ~3% of that from global non-glacial rivers (1,172,786 Gg year^−1^; Table 1). However, we estimate it is currently higher (3.9–9.5 times) than the combined GIS and AIS, even though total runoff^70^ is only 1.6 to 2.4 times higher (Fig. 5c and Table 1).

Our estimates contain substantial uncertainties, especially in the absence of catchment discharge-weighted mean cation concentration data from glaciers worldwide.

(i) The largest source of uncertainty is the scarcity of data on cation concentration from non-ice sheet glaciers outside of Asia (43 glaciers and 3561 samples in Asia versus 20 glaciers and 1080 samples in the rest of the world; Fig. 1A and Table 1).
(ii) There is considerable regional variability and the data assembled here do not allow for any statistically valid interpretation about how glacier size, sampling location/season/duration, and sample size at basins with similar bedrock as well as diverse geology spatially influence the cation concentration. The timing and duration of sampling may result in large errors owing to the high fluctuation of seasonal cation concentrations and/or meltwater runoff^26,45,46,57,61^. For example, daily mean Mg^2+^ concentration during 7–8 June is 9.4 times higher than during 8–9 August of 2013 at Dongkemadi Glacier in the Tanggula^26^. Monthly mean concentration of Mg^2+^ during July is 0.8 times lower than during an entire melt season (June to September) of 2013 at Urumqi Glacier No.1 in the Tianshan^45^.
(iii) Proglacial chemical weathering occurs rapidly and may play an important role in the cation concentration and export^26,79–81^, but the distance of 80% of sampling sites is below 2 km from ice margin in the current global data set (Supplementary Fig. 4) suggesting that the proglacial weathering will have relatively minor effects on cation exports in most cases.
(iv) If only the mid-summer data (*n* = 593) is used from 24 non-ice sheet glaciers worldwide, the estimated glacier cation flux is ~64% of that using all data (*n* = 4641) from all non-ice sheet glaciers, which is lower but does not significantly change the conclusions of this study (Fig. 1A and Supplementary Tables 8, 9).
(v) Geochemical data is currently not available for the large glaciers in Patagonia and Southern Andes (Fig. 1A). Although total runoff is comparatively small compared to global glacier runoff, glaciers in these regions are among the most rapidly retreating on Earth, and are likely to be regionally important in cation denudation.

Despite these reservations, our findings provide evidence that glaciers play a key role in catchment geochemical weathering and associated elemental mobilization.

### Glacial chemical weathering rates

Chemical weathering rates for glaciers within Asia appear to be controlled by several variables. Glacial weathering in the Altai is impacted by a very high discharge (3.63 m year^−^^1^)^82,83^, and the high observed Ca^2+^ concentration (750 μeq L^−^^1^) is the main component of the very high CDR (3425 Σ*meq^+^ m^−^^2^ year^−^^1^) (Fig. 5d and Table 1). The lowest CDR of 486 ± 105 Σ*meq^+^ m^−^^2^ year^−^^1^ is found in the Hengduan, where the specific discharge (0.65 m year^−^^1^) and cation concentration are relatively low. Higher CDR from the Himalayas (2000 ± 1586 Σ*meq^+^ m^−^^2^ year^−^^1^; Fig. 5d) is also consistent with a positive relationship between CDRs and specific discharge (Table 1). Lower CDRs and specific discharge along with slow glacier mass loss for mountain ranges on the Tibetan Plateau^84,85^ is further evidence of a link between meltwater generation and chemical weathering rates. On a regional scale, glacial CDR for Asia (2655 ± 2565 Σ*meq^+^ m^−^^2^ year^−^^1^) is similar to Arctic Canada, Svalbard and Jan Mayen, Alaska, Western Canada and USA, Central Europe and Iceland, and in the intermediate range of regional CDRs (285 to 9683 Σ*meq^+^ m^−^^2^ year^−^^1^; Fig. 5e and Table 1).

**Fig. 6:**
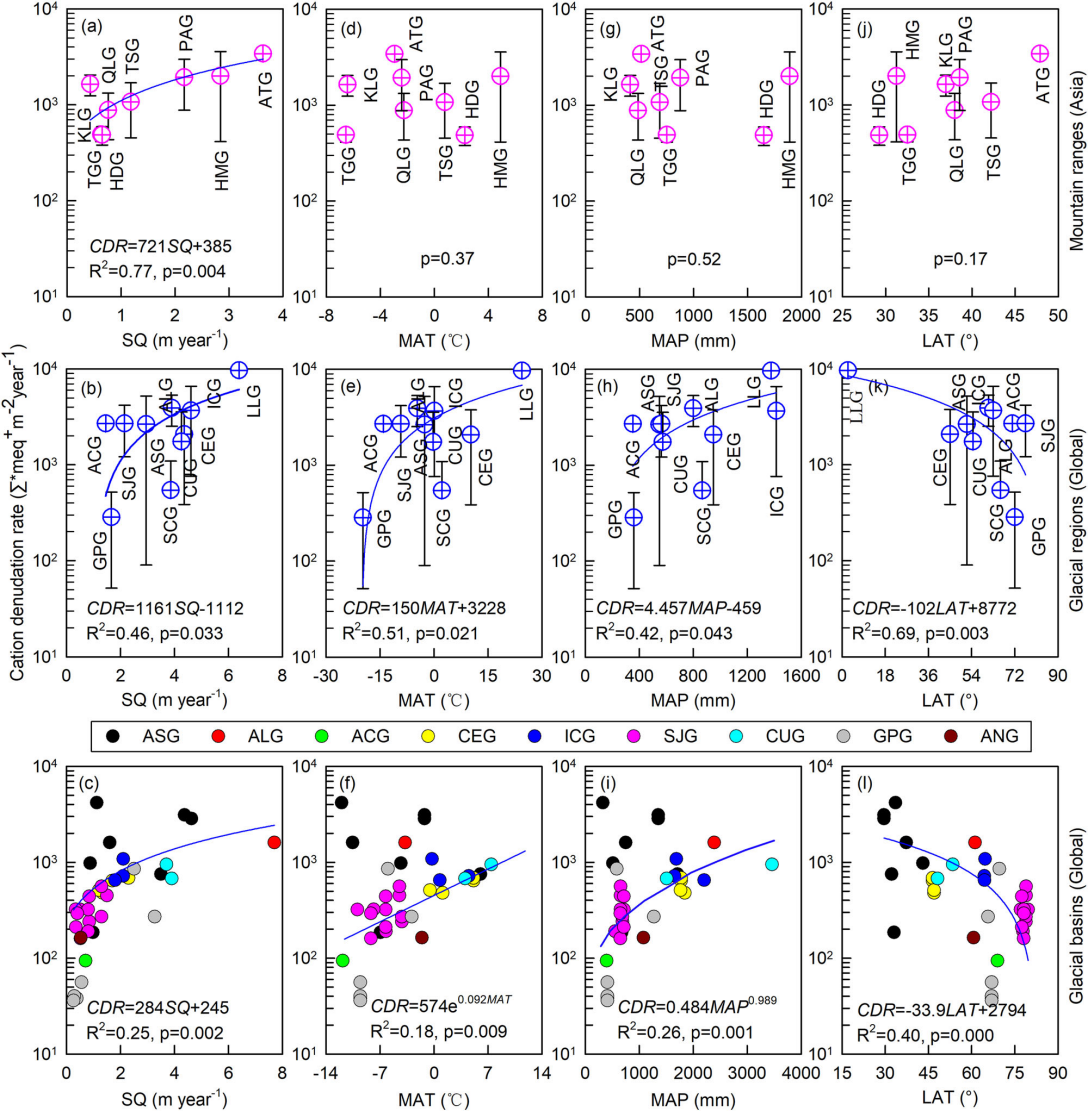
Relationships between cation denudation rate (CDR) and specific discharge (SQ), mean annual air temperature (MAT), mean annual precipitation (MAP), and the latitude (LAT) for glaciers globally. **a**, **d**, **g**, **j** Glaciers in eight mountain ranges within Asia; **b**, **e**, **h**, **k** Glaciers in ten glacial regions (excluding ANG; Table 1); **c**, **f**, **i**, **l** Glaciers at more than 29 glacial basins worldwide (Supplementary Table 10). Note that the horizontal lines above/below the symbols indicate the standard deviation.

Our data indicate that regional variability in glacial chemical weathering rates is related to the hydrological regime. We find a positive linear relationship between glacial CDRs and specific discharge on both mountain range (for eight mountain ranges in Asia; *R*^2^ = 0.77, *p* < 0.01) and regional (for ten glacial regions worldwide; *R*^2^ = 0.46, *p* < 0.04) scales (Fig. 6a, b). The highest CDR of 9683 Σ*meq^+^ m^−^^2^ year^−^^1^ is estimated for glaciers in the low latitude region, where the specific discharge is very high (6.39 m year^−^^1^). The lowest regional glacial CDR of 285 ± 233 Σ*meq^+^ m^−^^2^ year^−^^1^ we estimated occurs in Greenland Periphery (with a specific discharge of 1.66 m year^−^^1^; Fig. 5e and Table 1). The disproportionately high denudation rates from low latitude glaciers reflect high rates of mass loss^50,70,86^ as well as potentially higher rates of physical erosion compared to glaciers at high latitudes^14,87^, creating an abundance of reactive mineral surfaces.

Glacial cation denudation rates in glacial catchments appear to be globally elevated. Current CDR from glaciers globally (2174 ± 977 Σ*meq^+^ m^−^^2^ year^−^^1^) is ~3 times higher than previous mean glacial CDR (740 ± 830 Σ*meq^+^ m^−^^2^ year^−^^1^, see above). Furthermore, glacial CDR is ~3 times higher than the mean cation yield [~800 meq^+^ m^−^^2^ year^−^^1^ (380–1233 meq^+^ m^−^^2^ year^−^^1^), which is estimated by the discharge-weighted cation yield of 14.8 tons km^−^^2^ year^−^^1^] for Greenland Ice Sheet catchments (~150 to 2000 meq^+^ m^−^^2^ year^−^^1^, which is estimated by 2.6–37.6 tons km^−^^2^ year^−^^1^ from Leverett Glacier, Watson River, Proglacial zone of Watson River, Kiattuut Sermiat and “N” Glacier)^25^, and is approaching two orders of magnitude higher than CDR when compared to the whole ice sheet (29.5–43.1 Σ*meq^+^ m^−^^2^ year^−^^1^; Fig. 5f and Table 1). Glacial CDR is also ~4 times higher than the average CDR (~500 Σ*meq^+^ m^−^^2^ year^−^^1^) for the major world’s rivers (~200 to 1000 Σ*meq^+^ m^−^^2^ year^−^^1^ from Kolyma, Mackenzie, Ob, Nile, and Mississippi rivers)^88^ and ~6 times higher than the global river mean CDR (see above)^16,41–43^.

CDRs from glacial basins worldwide also show a linear relationship to specific discharge on a basin scale (for >29 glacial basins globally; *R*^2^ = 0.25, *p* < 0.01; Fig. 6c and Supplementary Table 10). Glacial CDRs are positively related to mean annual temperature (0.18 < *R*^2^ < 0.51, *p* < 0.03) and mean annual precipitation (0.26 < *R*^2^ < 0.42, *p* < 0.05) on both basin and regional scales (Fig. 6e, f, h, i). This indicates that climate warming driven meltwater runoff increase^50,51^ is a key control of glacier chemical denudation. The inverse CDR response to increasing latitude on both basin and regional scales (0.40 < *R*^2^ < 0.69; *p* < 0.01; Fig. 6k, l and Supplementary Table 10) is further evidence of the link between climatic warming, meltwater generation (specific discharge), and chemical weathering rates. This suggests that cation export will increase as glacier and ice sheet melt accelerates, while it will decrease as glaciers and/or ice sheets are removed from the landscape (or reaches a critical threshold). Geographically widespread studies are needed to further constrain estimates and help further address these relationships. Over the next few decades, non-ice sheet glaciers are likely to become increasingly important in chemical weathering budgets and related regional elemental cycles. This study highlights the role of glaciers on the catchment scale chemical weathering rates and emphasizes the poorly understood biogeochemical consequences of waning glacier cover with predicted warming of the Earth’s climate.

## Methods

### Study area

Fieldwork was conducted at the 19 glaciers in Asia, including four glaciers (KOG, DGG, BGG, and HGG) in the Tianshan, six glaciers (SG2, SG3, SG4, YG1, YG5, and LHG) in the Qilian, two glaciers (MKG, and YZG) in the Kunlun, one glacier (DKG) in the Tanggula, three glaciers (GG1, GG2, and GG3) in the Pamir, two glaciers (HLG, and HG2) in the Hengduan, and one glacier (RBG) in the Himalayan mountain ranges (Figs. 1B, and Supplementary Fig. 5 and Tables 1, 2). In this area, the regional climate is influenced by Indian monsoon and westerlies, with limited influence from East Asian monsoon on the Tibetan Plateau. In addition, mineral compositions of proglacial deposits at KOG are dominated by potash feldspar, quartz, and plagioclase (93.4%), followed by illite, calcite, dolomite, kaolinite, and siderite (6.4%), and chlorite, pyrite, and gypsum (0.2%) (Supplementary Table 6). This is similar to the dominance of mineral compositions at DKG in the Tanggula^26^, at HLG in the Hengduan^46^, at Urumqi Glacier No.1 in the Tianshan^45^, and at Qiyi Glacier in the Qilian^60^ within Asia.

### Sampling and laboratory analysis

Glacier outflows from the 19 Asian glaciers were sampled manually at locations as close to ice margin as possible during fieldwork that is supported by the PRC Ministry of Science and Technology (Supplementary Fig. 5). The distance of sampling sites from ice margin ranged from 10 to 100 s of meters, depending on the confluence conditions and the accessibility of meltwater river channels. Bulk meltwater samples (*n* = 591) were taken every hour over a 1–4 day period in July–October of 2007 or April–August of 2008 to observe the diurnal process of cation concentrations and relevant drainage system in glacial environments (Supplementary Tables 2 and 3). Before sampling, precleaned bottles were rinsed three times in situ by river water. In 2013, glacial deposits (*n* = 10; diameter is below 5 mm) were sampled as close to the ice margin and river channels as possible in the proglacial area at KOG to characterize their mineral compositions (Supplementary Table 7). After collection samples were immediately frozen and transported to the State Key Laboratory of Cryospheric Science of the Chinese Academy of Sciences for chemical analysis. Samples were immediately filtered through 0.45 μm cellulose nitrate membranes after melting at room temperature (Supplementary Table 1). Concentrations of total dissolved solids were determined with a precision of ±1% using a CON410 meter. Major dissolved cations (Na^+^, K^+^, Mg^2+^, and Ca^2+^) were determined by ion chromatography using a Dionex-600 fitted with an IonPac CS-12A-HC column, using 20 mM MSA eluent and a CSRS-ULTRA-II suppressor. The detection limits for all ions were below 0.85 μeq L^−^^1^ and the precision was better than ±1% for all measured ions. The comparative experiments on some samples indicated that the influence of the frozen-melt process after sampling on cation concentrations is negligible. The mineral compositions were determined by X-ray diffraction (D/Max-IIIB) using a copper butt, step-continuous scanning with 4°/min of speed, and tube 40 kV and 25 mA^26,60^.

### Compiled data set for cations and glacial runoff

Published cation concentrations for meltwater runoff (*n* = 4874) were compiled from 62 glaciers in eleven glacial regions (Fig. 1A and Supplementary Table 3). This data set includes 28 glaciers located in Asia (ASG), 2 glaciers in Alaska (ALG), 1 glacier in Arctic Canada (ACG), 5 glaciers in Central Europe (CEG), 2 glaciers in Iceland (ICG), 1 glacier in Low Latitudes (LLG), 1 glacier in Scandinavia (SCG), 4 glaciers in Svalbard and Jan Mayen (SJG), 4 glaciers in Western Canada and USA (CUG), 9 outlet glaciers in Greenland Periphery (GPG), and 5 outlet glaciers in Antarctic and Subantarctic (ANG; Supplementary Tables 1 and 3). This data set is combined with new data from the 19 glaciers (see above) in eight mountain ranges (ATG, TSG, QLG, KLG, TGG, PAG, HDG, and HMG) within Asia generated in this study to produce a global data set (where cations were not corrected for the sea-salt and aerosol contributions) containing 5465 samples from 77 glaciers (including 14 ice sheet outlet glaciers) worldwide (Fig. 1A and Supplementary Table 3).

Modeled annual glacial runoff for eight mountain ranges within Asia during 2000–2014 was taken from Wang^82^, and the modeled runoff for other ten glacial regions (excluding ANG; see above) and global glaciers (Table 1) during 2003–2022 were taken from Bliss and others^70^. In brief, annual glacial runoff from Asian mountain ranges was calculated based on a glacier mass balance model and a glacier runoff model. The mass balance model was run on a monthly timescale^89,90^, driven by monthly precipitation and air temperature. For the debris-free glaciers, ice surface ablation was calculated based on a degree-day model. But for the debris-covered glaciers, an ablation influencing factor was introduced to the degree-day model based on a relationship between the debris thickness and sub-ice ice melt rates^91^. The runoff model was proposed by Bliss and others^70^, in which the evaporation, sublimation, and any storage changes (groundwater, englacial water, and subglacial water) were assumed to be negligible^92^. Notably, glacier mass balance is the basis of the runoff model, and all variables involved are integrated over an entire glacier-covered area. The same input data, calibrated parameters for the mass balance model were utilized to drive the glacier runoff model, and the observations from 45 monitored glaciers were used to calibrate related parameters^93^, following the method of Radic and Hock^89,90^. To improve the accuracy of modeling results, the ASTER DEMs-derived geodetic glacier mass balance from Brun and others^94^ were used to further constrain the modeled results. In detail, the glacier mass balance and glacier runoff models are briefly illustrated below.

#### Glacier mass balance model

The monthly-scale mass balance model^89,90^ was used in this study to model glacier mass balance for mountain ranges in High Mountain Asia during 1952–2014. In detail, the primary input data involved in this model includes monthly precipitation and air temperature. Glacier area-weighted specific mass balance (*B*) for the whole glacier in each mountain range was computed as a sum of specific mass balance of each elevation band on a glacier.

1 $$B=\frac{{\sum }_{i=1}^{n}{b}_{i}\times {S}_{i}}{{\sum }_{i=1}^{n}{S}_{i}}$$

where *b*_*i*_ and *S*_*i*_ denote specific mass balance and glacier area, respectively, and subscripts (*i*) represent the number of elevation band on a glacier (*i* = 1, 2, 3, …, *n*).

Monthly specific mass balance (*b*_*i*_) of each elevation band on a glacier (with an interval of 50 m) was calculated as

2 $${b}_{i}={a}_{i}+{c}_{i}+{R}_{i}$$

where *a*_*i*_ denotes glacier surface ablation (negative), *c*_*i*_ represents glacier mass accumulation (positive), and *R*_*i*_ refers to snowmelt refreezing (positive) at each elevation band.

For debris-free glaciers, the glacier surface ablation (*a*_*i*_) was calculated based on a degree-day model, in which snow/ice melt is considered as a linear correlation to monthly air temperature. Here, monthly *a*_*i*_ (mm w.e.) was expressed as

3 $${a}_{i}={f}_{{{{{{\mathrm{snow}}}}}}/{{{{{\mathrm{ice}}}}}}}\times \int \max ({T}_{i},0){{{{{\mathrm{d}}}}}}t$$

where *f*_snow/ice_ denotes degree-day factor for snow/ice (mm w.e. d^−^^1^ °C^−^^1^), and *T*_*i*_ denotes monthly air temperature (°C) above the glacier surface.

For debris-covered glaciers, the relationship between debris thickness and glacier surface ablation^91^ was used to calculate the effect of debris cover on glacier surface ablation. In detail, an averaged curve for debris thickness and surface ablation was used in the mass balance model to calculate the reduction of ice ablation due to debris cover. To calculate the ablation of debris-covered glaciers, the ablation factor *k*_*i*_ was introduced to quantify the effect of debris on glacier surface ablation. Thus, monthly glacier surface ablation due to debris cover (*a*_*i*,debris_) was calculated as

4 $${a}_{i,{{{{{\mathrm{debris}}}}}}}={k}_{i}\times {f}_{{{{{{\mathrm{snow}}}}}}/{{{{{\mathrm{ice}}}}}}}\times \int \max ({T}_{i},0){{{{{\mathrm{d}}}}}}t$$

where *k*_*i*_ denotes the scale factor of each elevation band (*i*) which depends on the debris thickness.

Monthly glacier mass accumulation for each elevation band *c*_*i*_ (mm w.e.) was calculated as

5 $${c}_{i}={\delta }_{m}\times {P}_{i}\left\{\begin{array}{c}\kern-6pt{\delta }_{m}=1,{T}_{i} \, < \, {T}_{{{{{{\mathrm{snow}}}}}}}\\ \kern-3pt{\delta }_{m}=0,{T}_{i}\ge {T}_{{{{{{\mathrm{snow}}}}}}}\end{array}\right.$$

where *δ*_*m*_ is a constant, *T*_*i*_ denotes air temperature at each elevation band, and *T*_snow_ represents the threshold temperature. If *T*_*i*_ is below *T*_snow_, *P*_*i*_ is assumed to be snow; if not, *P*_*i*_ is regarded as liquid precipitation.

Based on the relationship between annual potential refreezing *R*_*i*,pot_ (cm) and air temperature *T*_*a*_ (°C) at each elevation band^95^, *R*_*i*,pot_ was calculated as

6 $${R}_{i,{{{{{\mathrm{pot}}}}}}}=-0.69\times {T}_{a}+0.0096$$

where the lower boundary of annual snowmelt refreezing over the whole glacier is zero, while an upper boundary is applied in the ablation zone and assumed equal to the accumulated snow. Monthly snow meltwater frozen on glacier surface (*R*_*i*_) does not flow away until accumulated melt in a mass balance year exceeds the annual potential refreezing (*R*_*i*,pot_).

To interpolate the Climatic Research Unit Time-Series (CRU TS) monthly temperature at each elevation band, two temperature lapse rates (*T*_lap_, and *G*_lap_) were utilized. *T*_lap_ is a parameter similar to a ‘statistical lapse rate’ between the CRU TS altitude of grid cell with glacier location (*h*_CRU_) and the highest elevation of glacier (*h*_max_), while *G*_lap_ denotes glacier surface temperature mainly taking into account glacier surface difference, such as orientation and glacier surface climate environment. Monthly air temperature at each elevation band (*T*_*i*_) was calculated as

7 $${T}_{i}={T}_{{{{{{\mathrm{CRU}}}}}}}+{T}_{{{{{{\mathrm{lap}}}}}}}\times ({h}_{\max }-{h}_{{{{{{\mathrm{CRU}}}}}}})+{G}_{{{{{{\mathrm{lap}}}}}}}\times (h-{h}_{\max })$$

where *T*_CRU_ denotes the CRU TS monthly air temperature during the period of 1952–2014, and *h* represents the altitude of glacier elevation band.

To interpolate the CRU TS monthly precipitation to *h*_max_, a precipitation correction factor (*k*_p_) was assigned, while a precipitation gradient (*d*_pre_) was used to interpolate precipitation to each elevation band (the percentage of precipitation decreases with every 50 m decreases in elevation) from the highest to lowest elevation of the glacier. Thus, monthly precipitation at each elevation band was calculated as

8 $${P}_{i}={k}_{{{{{{\mathrm{p}}}}}}}\times {P}_{{{{{{\mathrm{CRU}}}}}}}\times \lfloor 1+{d}_{{{{{{\mathrm{pre}}}}}}}\times (h-{h}_{\max })\rfloor$$

where *P*_CRU_ denotes CRU TS monthly grid precipitation for 1952–2014 at the cell of the glacier located.

#### Glacier runoff model

According to the method proposed by Bliss and others^70^, glacier runoff was calculated using a water balance approach. For individual glaciers in mountain range within High Mountain Asia during 1952–2014, annual glacier runoff (*Q*_*i*_) at each elevation band on a glacier was calculated as:

9 $${Q}_{i}=\mathop{\sum }\limits_{i=1}^{n}{S}_{i}\times ({a}_{i}+{P}_{{{{{{\mathrm{liq}}}}}},i}-{R}_{i})$$

where *a*_*i*_ denotes the melt of ice, firn and snow, as in Eq. (3), which is meltwater from glacier net mass loss. *P*_liq,*i*_ represents liquid precipitation as in Eq. (8). Notably, if *T*_m_ is above the threshold temperature (*T*_snow_), *P*_*i*_ is regarded as liquid precipitation. *R*_*i*_ refers to potential refreezing at each elevation band as in Eq. (6). *S*_*i*_ indicates glacier area of each elevation band *i*.

### Cation flux estimates

To calculate the annual cation fluxes from eight mountain ranges and nine non-ice sheet glacial regions (Fig. 1) we used mean annual runoff^70,82^ multiplied by data set mean Na^+^, K^+^, Mg^2+^, and Ca^2+^ concentrations to produce annual Na^+^, K^+^, Mg^2+^, and Ca^2+^ fluxes (Table 1 and Supplementary Table 5). These were used to generate cation fluxes for each mountain range or glacial region (Table 1). Annual cation flux from all glaciers worldwide was estimated by two methods: (i) the discharge extrapolation of annual Na^+^, K^+^, Mg^2+^, and Ca^2+^ fluxes from nine non-ice sheet glacial regions based on the percentage of annual glacial runoff from these nine glacial regions to annual glacial runoff globally^70^ and then the extrapolated annual Na^+^, K^+^, Mg^2+^, and Ca^2+^ fluxes were summed, and (ii) the multiplication of regional discharge-weighted mean Na^+^, K^+^, Mg^2+^, and Ca^2+^ concentrations (as the mean cation concentrations for glaciers worldwide) by annual glacial runoff globally^70^ and then annual Na^+^, K^+^, Mg^2+^, and Ca^2+^ fluxes were summed (Table 1 and Supplementary Table 8). In order to evaluate the effect of discharge-concentration seasonality on estimated cation flux from global glaciers, annual cation flux from global glaciers was also estimated by the discharge-weighted method as described above based on the mid-summer (July–August) data (*n* = 593) from 24 glaciers worldwide in six non-ice sheet glacial regions (ASG, CEG, ICG, LLG, SCG, and CUG; Supplementary Tables 8 and 9). Similar extrapolations have been adopted in recent estimates of element flux from glaciers and/or ice sheets^10–12,26,66^. The approaches are simplistic but provide a first-order estimate of cation fluxes on the mountain range and regional scales. The approaches taken assume that glaciers with available data are representative of entire mountain ranges or glacial regions, which is unlikely given the differences in geology, glacial hydrology, and temporal variation in sampling. Error estimates of cation fluxes were calculated from the standard deviation of data set mean cation concentrations multiplied by annual glacial runoff (Table 1).

### Cation denudation rate estimates

To calculate the crustal CDRs we use the annual *Na^+^, *K^+^, *Mg^2+^, and *Ca^2+^ fluxes divided by glacial area^11,12^ (asterisks indicate crustal-derived solutes; Table 1). The annual *Na^+^, *K^+^, *Mg^2+^ and *Ca^2+^ fluxes were estimated by the annual Na^+^, K^+^, Mg^2+^, and Ca^2+^ fluxes as described above multiplied by the mean contribution (%) of the annual *Na^+^, *K^+^, *Mg^2+^, and *Ca^2+^ fluxes to the annual total Na^+^, K^+^, Mg^2+^, and Ca^2+^ fluxes which is calculated based on published data from 2 glaciers in Asia, more than 8 glaciers in Svalbard, 1 glacier in Central Europe and 1 outlet glacier in Greenland for each mountain range or glacial region (Table 1). In detail, for eight mountain ranges in Asia and the Asian glacial region, the mean contributions of the annual *Na^+^, *K^+^, *Mg^2+^, and *Ca^2+^ fluxes to the annual total Na^+^, K^+^, Mg^2+^, and Ca^2+^ fluxes were calculated based on the contributions of crustal-derived cations to total cations at Batura Glacier^27^ and Hailuogou Glacier^32^. For the SJG, the mean contributions were calculated based on the crustal-derived cation contributions at Scott Turnerbreen^28^, Austre Brøggerbreen^24^, Erdmannbreen^24^, Midre Lovénbreen^24^, Hannabreen^24^, Erikbreen^24^, Longyearbreen^30^, and Bayelva River^29^ in Svalbard. For the CEG, the mean contributions were calculated based on the crustal-derived cation contributions at Haut Glacier d’Arolla^21^ in Central Europe. For the GIS and AIS, the mean contributions were calculated based on the crustal-derived cation contributions at Kuannersuit Glacier^31^ in the Greenland. For the other six glacial regions (ALG, ACG, ICG, LLG, SCG, and CUG) and global glaciers, the mean contributions were calculated based on the crustal-derived cation contributions at all non-ice sheet glaciers (apart from Kuannersuit Glacier) because data are not available in these six regions (Table 1).

## Supplementary information

Supplementary Information

## Data Availability

All data used in this study are present in supplementary materials and have been deposited in the National Tibetan Plateau/Third Pole Environment Data Center (http://data.tpdc.ac.cn/en/disallow/5a1eb9ee-462a-4840-af81-98caf3738665/).
