# Supplementary material for: Globally elevated chemical weathering rates beneath glaciers
Source: Nat Commun. 2022 Jan 20;13:407. doi: 10.1038/s41467-022-28032-1 (PMC8776776; doi:10.1038/s41467-022-28032-1)
Supplement: Supplementary file 1 — Supplementary Information [file 41467_2022_28032_MOESM1_ESM.pdf]

Globally elevated chemical weathering rates beneath glaciers

Supplementary Information

Xiangying Li[1,2,3,†,✉], Ninglian Wang[1,2,4,†,✉], Yongjian Ding[5,6,7,†,✉], Jon R. Hawkings[8], Jacob C. Yde[9], Robert Raiswell[10], Jintao Liu[11], Shiqiang Zhang[1-2], Shichang Kang[5-6], Rongjun Wang[5], Qiao Liu[12], Shiyin Liu[13], Roland Bol[14-15], Xiaoni You[16], Guoyu Li[3,6]

[1] Shaanxi Key Laboratory of Earth Surface System and Environmental Carrying Capacity, Northwest University, Xi'an, China.
[2] College of Urban and Environmental Sciences, Northwest University, Xi'an, China.
[3] State Key Laboratory of Frozen Soil Engineering, Northwest Institute of Eco-Environment and Resources, Chinese Academy of Sciences, Lanzhou, China.
[4] CAS Center for Excellence in Tibetan Plateau Earth Sciences, Beijing, China.
[5] State Key Laboratory of Cryospheric Science, Northwest Institute of Eco-Environment and Resources, Chinese Academy of Sciences, Lanzhou, China.
[6] University of Chinese Academy of Sciences, Beijing, China.
[7] China-Pakistan Joint Research Center on Earth Sciences, CAS-HEC, Islamabad, Pakistan.
[8] Department of Earth and Environmental Science, University of Pennsylvania, Hayden Hall, 240 South 33rd Street, Philadelphia, USA.
[9] Department of Environmental Sciences, Western Norway University of Applied Sciences, Sogndal, Norway.
[10] Cohen Biogeochemistry Laboratory, School of Earth and Environment, University of Leeds, Leeds LS2 9JT, UK.
[11] College of Hydrology and Water Resources, Hohai University, Nanjing, China.
[12] Institute of Mountain Hazards and Environment, Chinese Academy of Sciences, Chengdu, China.
[13] Institute of International Rivers and Eco-security, Yunnan University, Kunming, China.
[14] Forschungszentrum Jülich IBG-3, Wilhelm-Johnen-Straße, Jülich, Germany.
[15] School of Natural Sciences, Environment Centre Wales, Bangor University, Bangor, LL57 2UW, UK.
[16] College of Resources and Environmental Engineering, Tianshui Normal University, Tianshui, China
[†] These authors contributed equally: Xiangying Li, Ninglian Wang, Yongjian Ding.
[✉] email: shaanxilxy@163.com; nlwang@nwu.edu.cn; dyj@lzb.ac.cn

The supplementary files include 5 figures and 10 tables.

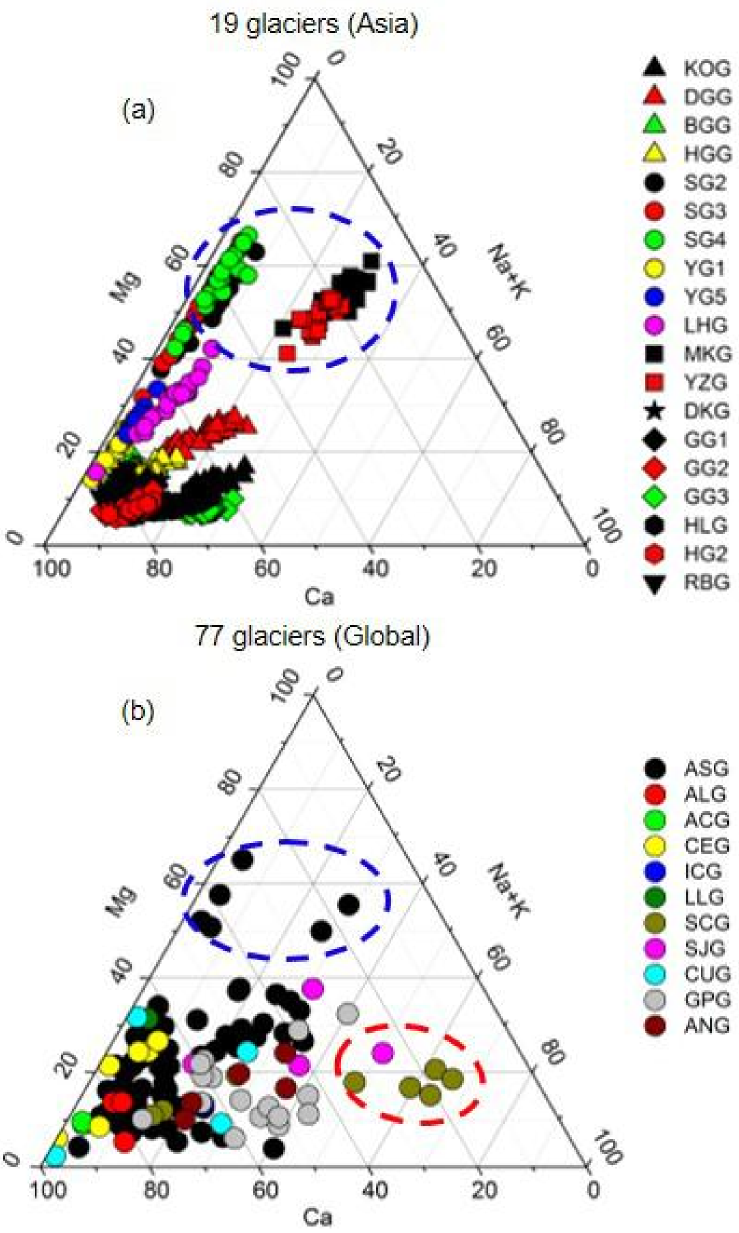

Supplementary Figure 1: Piper plots showing the abundance of major cations (μeq L^{-1}) from glaciers globally. **a** 19 glaciers in Asia generated in this study. **b** 77 glaciers worldwide in eleven glacial regions including 43 glaciers in Asia (ASG), 2 glaciers in Alaska (ALG), 1 glacier in Arctic Canada (ACG), 5 glaciers in Central Europe (CEG), 2 glaciers in Iceland (ICG), 1 glacier in Low Latitudes (LLG), 1 glacier in Scandinavia (SCG), 4 glaciers in Svalbard and Jan Mayen (SJG), 4 glaciers in Western Canada and USA (CUG), 9 outlet glaciers in Greenland Periphery (GPG), and 5 outlet glaciers in Antarctic and Subantarctic (ANG). Note that data within the blue dashed circles denote SG2, SG3, SG4, MKG, YZG and Gangotri Glacier in ASG, within the red dashed circles denote Scott Turnerbreen in SJG and Austre Okstindbreen in SCG (Supplementary Tables 1-3).

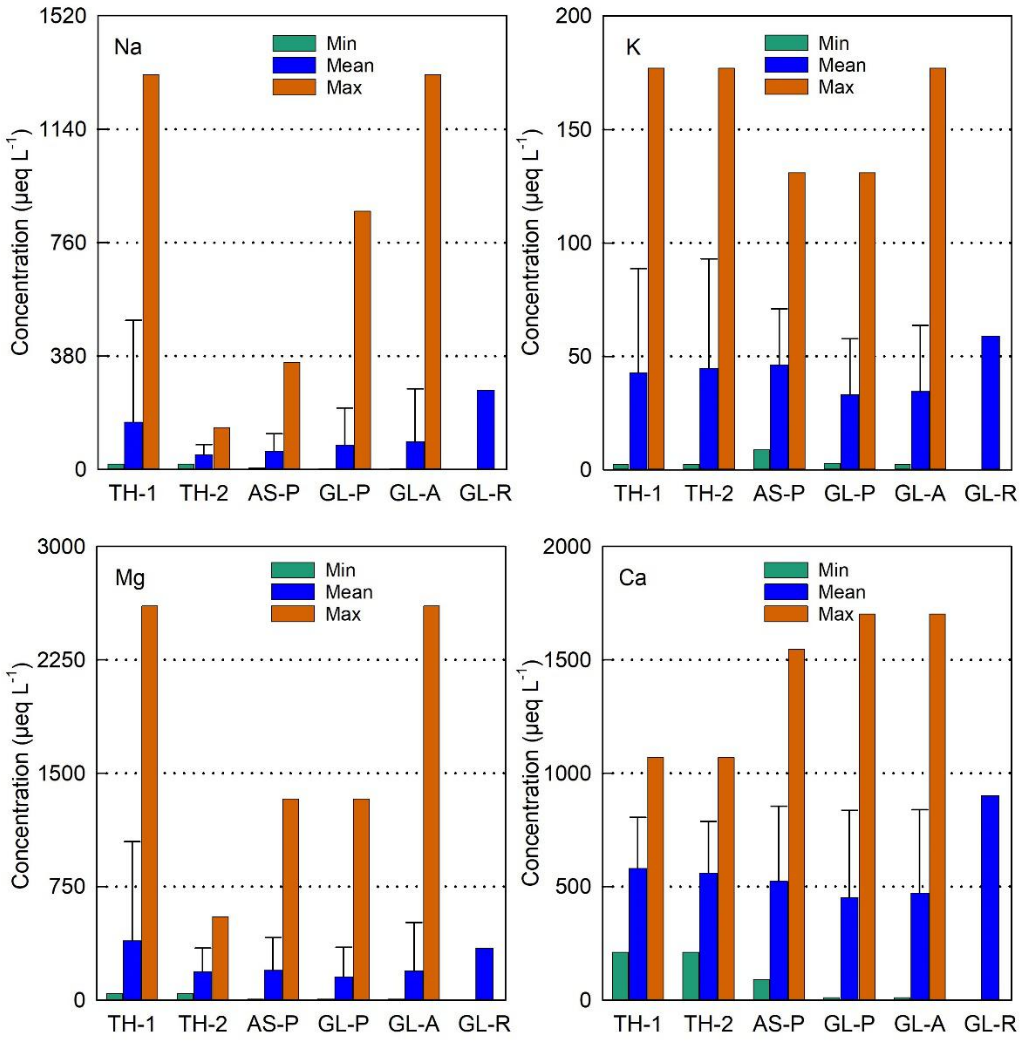

Supplementary Figure 2: Comparison of minimum (Min), mean (Mean) and maximum (Max) concentrations of Na^{+}, K^{+}, Mg^{2+} and Ca^{2+} from glaciers with global non-glacial rivers. TH-1, TH-2, AS-P, GL-P, GL-A and GL-R denote the 19 glaciers generated in this study, the 17 of 19 glaciers generated in this study (apart from Meikuang Glacier and Yuzhufeng Glacier in the Kunlun), previously published 28 glaciers in Asia, previously published 63 glaciers globally, all 77 glaciers in current global dataset (Supplementary Table 3), and global non-glacial rivers[1,2] respectively. Note that the horizontal lines above the bar plots indicate the standard deviation.

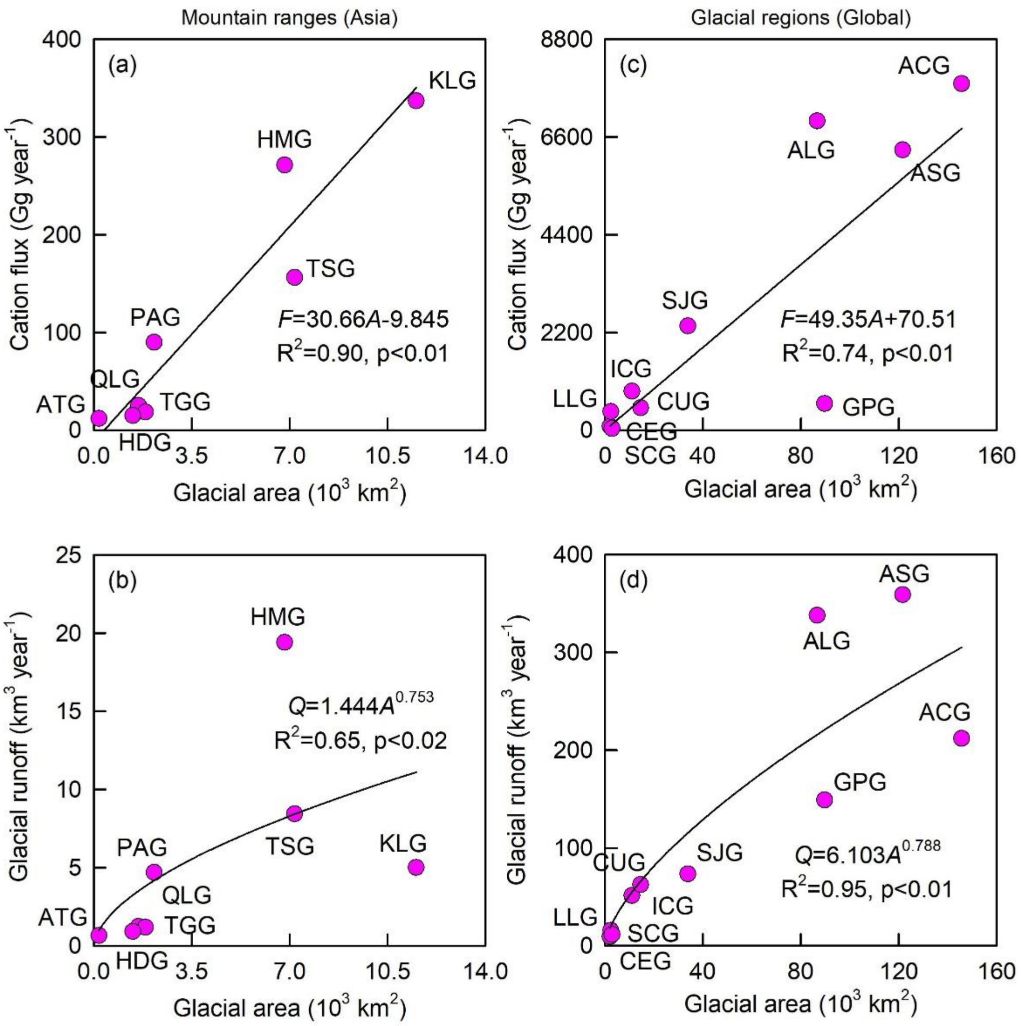

Supplementary Figure 3: Relationships between glacial area (*A*) and cation flux (*F*) and glacial runoff (*Q*). a-b Glaciers in eight mountain ranges within Asia including the Altai (ATG), the Tianshan (TSG), the Qilian (QLG), the Kunlun (KLG), the Tanggula (TGG), the Pamir (PAG), the Hengduan (HDG), and the Himalayan (HMG). **c-d** Glaciers in ten glacial regions worldwide including Asia (ASG), Alaska (ALG), Arctic Canada (ACG), Central Europe (CEG), Iceland (ICG), Low Latitudes (LLG), Scandinavia (SCG), Svalbard and Jan Mayen (SJG), Western Canada and USA (CUG), and Greenland Periphery (GPG). Note that cation flux is closely correlated to glacial area on both mountain-range and regional scales.

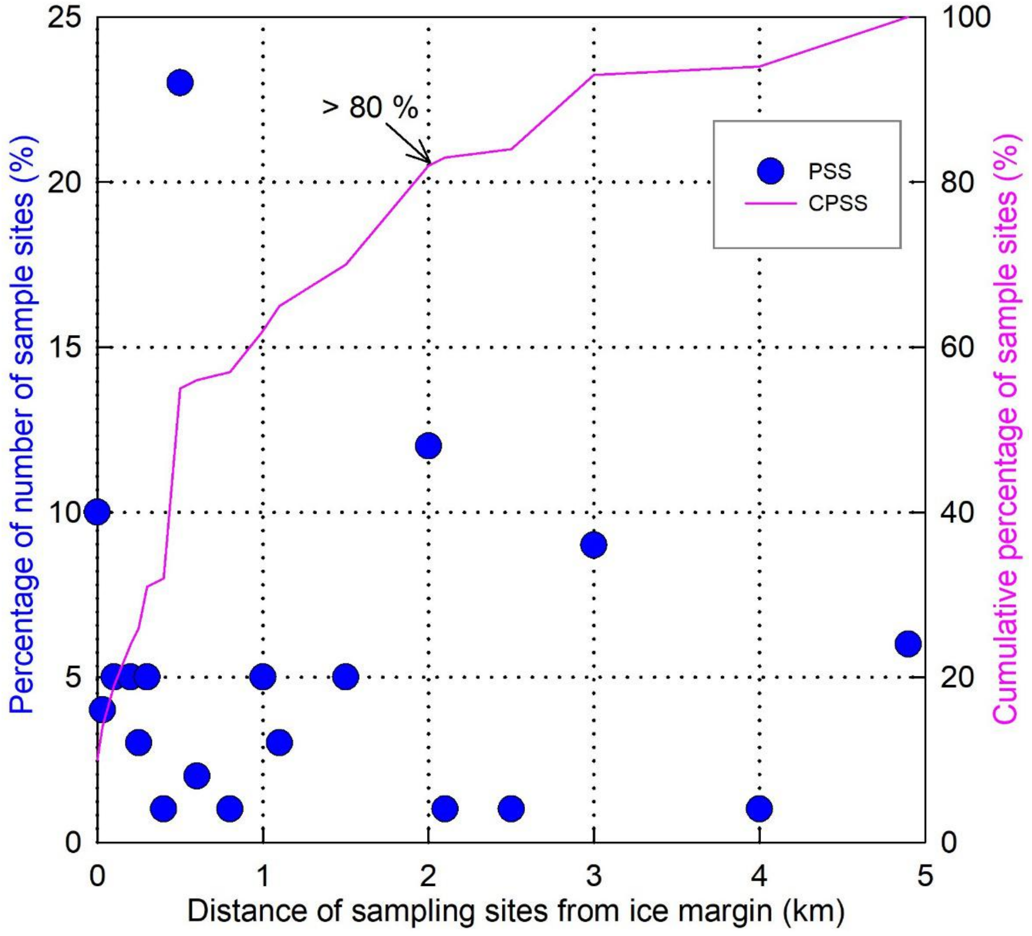

Supplementary Figure 4: The percentage (PSS) and cumulative percentage (CPSS) of the number of sampling sites with different distances from ice margin accounting for total sampling sites for glaciers in eleven glacial regions worldwide (Supplementary Table 3). Note that some sampling sites overlapped at the same distance from ice margin, and the sampling sites with the distance less than 2 km from ice margin accounts for more than 80 % of total sampling sites.

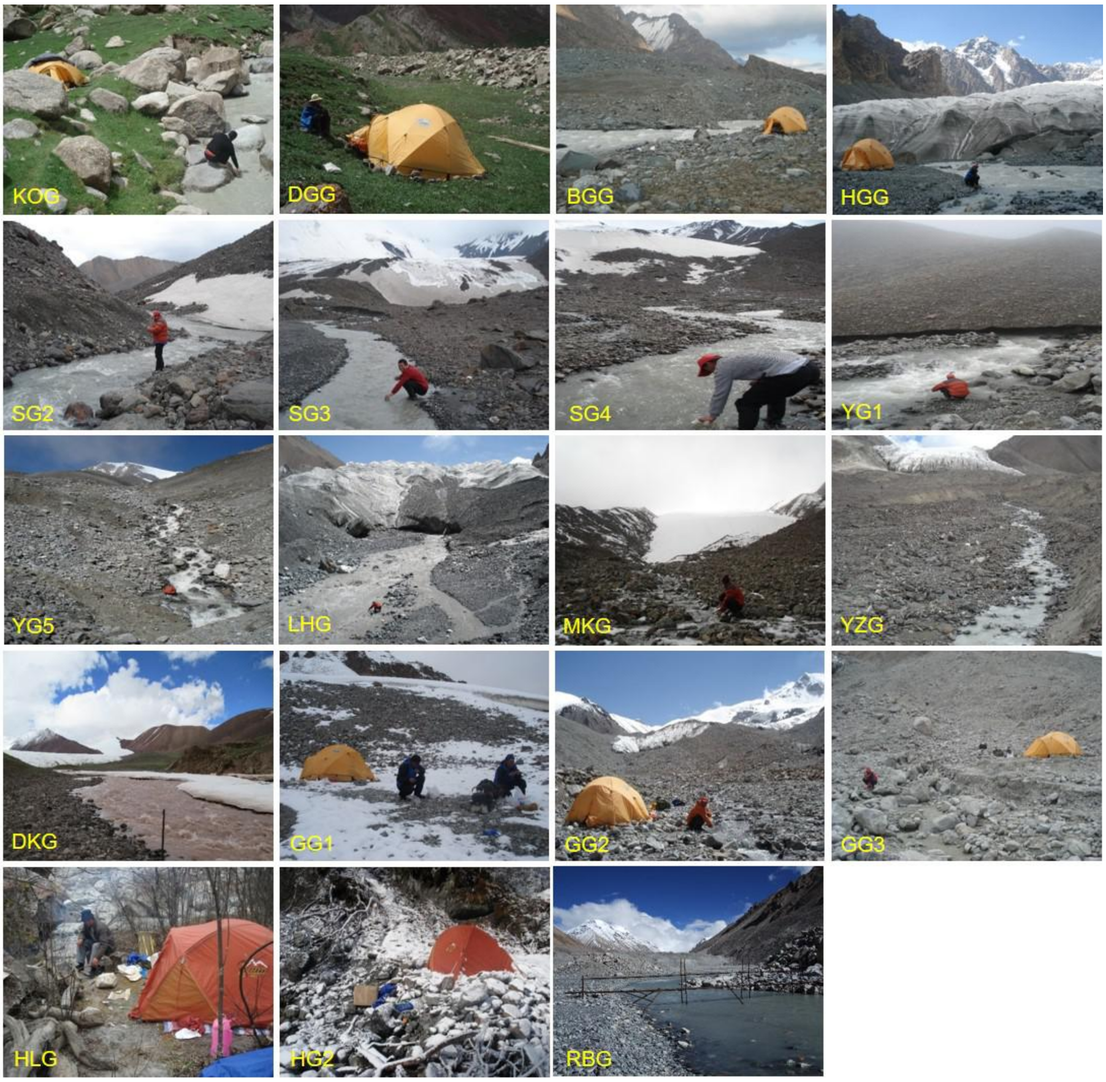

Supplementary Figure 5: Location of sampling sites for the 19 Asian glaciers generated in this study during July to October of 2007 or April to August of 2008. These glaciers include 4 glaciers (KOG, DGG, BGG, and HGG) in the Tianshan, 6 glaciers (SG2, SG3, SG4, YG1, YG5, and LHG) in the Qilian, 2 glaciers (MKG, and YZG) in the Kunlun, 1 glacier (DKG) in the Tanggula, 3 glaciers (GG1, GG2, and GG3) in the Pamir, 2 glaciers (HLG, and HG2) in the Hengduan, and 1 glacier (RBG) in the Himalayan mountain ranges (Supplementary Tables 1-2). Note that the distances of sampling sites from ice margin are less than 500 m for all glaciers.

Supplementary Table 1: Summary for the abbreviation (Abbr) of 19 Asian glaciers generated in this study, eight mountain ranges within Asia, and eleven glacial regions globally throughout this study. Note that the glacial regions worldwide (excluding Greenland and Antarctic ice sheets) were defined by Randolph Glacier Inventory (RGI) which is a collection of digital outlines of the world's glaciers[3,4].

Glacier	Abbr	Mountain range	Abbr	Glacial region	Abbr
Koxkar Glacier	KOG	Altai Mountains	ATG	Asia	ASG
Donggou Glacier	DGG	Tianshan Mountains	TSG	Alaska	ALG
Bogeda Glacier	BGG	Qilian Mountains	QLG	Arctic Canada	ACG
Heigou Glacier	HGG	Kunlun Mountains	KLG	Central Europe	CEG
Shuiguanhe Glacier No.2	SG2	Tanggula Mountains	TGG	Iceland	ICG
Shuiguanhe Glacier No.3	SG3	Pamir Mountains	PAG	Low Latitudes	LLG
Shuiguanhe Glacier No.4	SG4	Hengduan Mountains	HDG	Scandinavia	SCG
Yanglonghe Glacier No.1	YG1	Himalayan Mountains	HMG	Svalbard and Jan Mayen	SJG
Yanglonghe Glacier No.5	YG5			Western Canada and USA	CUG
Laohugou Glacier	LHG			Greenland Periphery	GPG
Meikuang Glacier	MKG			Antarctic and Subantarctic	ANG
Yuzhufeng Glacier	YZG				
Dongkemadi Glacier	DKG				
Gongger Glacier No.1	GG1				
Gongger Glacier No.2	GG2				
Gongger Glacier No.3	GG3				
Hailuogou Glacier	HLG				
Hailuogou Glacier No.2	HG2				
Rongbulk Glacier	RBG				

Supplementary Table 2: Summary for the longitude (LON; °) and latitude (LAT; °), distance of sampling sites from ice margin (km), sampling period, filter type (Filter) and pore size (Pore; µm) for the 19 Asian glaciers in seven mountain ranges (Mount) generated in this study. These glaciers include 4 glaciers in the Tianshan (TSG), 6 glaciers in the Qilian (QLG), 2 glaciers in the Kunlun (KLG), 1 glacier in the Tanggula (TGG), 3 glaciers in the Pamir (PAG), 2 glaciers in the Hengduan (HDG), and 1 glacier in the Himalayan (HMG).

Mount	Glacier	LON	LAT	Distance	Sampling period	Filter	Pore
TSG	KOG	80.17	41.81	0.3	2008/7/13 9:00 to 2008/7/14 15:00	CNM	0.45
	DGG	80.18	41.80	0.2	2008/6/21 15:00 to 2008/6/23 2:00	CNM	0.45
	BGG	88.27	43.80	0.5	2008/7/23 13:00 to 2008/7/26 12:00	CNM	0.45
	HGG	88.36	43.78	0.1	2008/8/1 11:00 to 2008/8/3 12:00	CNM	0.45
QLG	SG2	101.78	37.54	0.5	2007/7/8 8:00 to 2007/7/9 7:00	CNM	0.45
	SG3	101.78	37.54	0.3	2007/7/7 8:00 to 2007/7/8 7:00	CNM	0.45
	SG4	101.78	37.54	0.2	2007/7/3 8:00 to 2007/7/4 7:00	CNM	0.45
	YG1	98.59	39.23	0.1	2007/8/14 8:00 to 2007/8/15 8:00	CNM	0.45
	YG5	98.59	39.23	0.5	2007/8/14 11:00 to 2007/8/14 20:00	CNM	0.45
	LHG	96.55	39.47	0.1	2007/8/1 8:00 to 2007/8/2 7:00	CNM	0.45
KLG	MKG	94.19	35.68	0.1	2007/9/30 10:00 to 2007/10/1 9:00	CNM	0.45
	YZG	94.22	35.63	0.4	2007/10/8 10:00 to 2007/10/9 9:00	CNM	0.45
TGG	DKG	92.07	33.07	0.3	2007/10/17 9:00 to 2007/10/18 9:00	CNM	0.45
PAG	GG1	75.27	38.48	0.4	2008/6/8 14:00 to 2008/6/9 13:00	CNM	0.45
	GG2	75.05	38.67	0.3	2008/6/1 14:00 to 2008/6/2 14:00	CNM	0.45
	GG3	75.19	38.51	0.5	2008/6/6 13:00 to 2008/6/7 14:00	CNM	0.45
HDG	HLG	101.98	29.57	0.3	2008/4/15 10:00 to 2008/4/17 12:00	CNM	0.45
	HG2	101.97	29.55	0.5	2008/4/11 11:00 to 2008/4/13 11:00	CNM	0.45
HMG	RBG	86.83	28.18	0.5	2008/6/11 19:00 to 2008/6/12 23:00	CNM	0.45

Note. CNM denotes cllulose nitrate membrane.

Supplementary Table 3: Summary for the longitude (LON; °) and latitude (LAT; °), specific discharge (SQ; m year^{-1}), distance of sampling sites from ice margin (km), sampling period, filter type (Filter) and pore size (Pore; μm), as well as the mean concentrations of total dissolved solids (TDS; mg L^{-1}) and major cations (Na^+, K^+, Mg^{2+}, and Ca^{2+}; μeq L^{-1}) for 77 glaciers (63 mountain/valley glaciers and 14 ice sheet outlet glaciers) in eleven glacial regions worldwide. These glaciers include 43 glaciers in eight mountain ranges (Mount) within Asia (ASG; 1 glacier in the Altai, 6 glaciers in the Tianshan, 4 glaciers in the Pamir, 7 glaciers in the Qilian, 2 glaciers in the Kunlun, 1 glacier in the Tanggula, 2 glaciers in the Hengduan, and 20 glaciers in the Himalayan), and 34 glaciers in other ten glacial regions outside of Asia [2 glaciers in Alaska (ALG), 1 glacier in Arctic Canada (ACG), 5 glaciers in Central Europe (CEG), 2 glaciers in Iceland (ICG), 1 glacier in Low Latitudes (LLG), 1 glacier in Scandinavia (SCG), 4 glaciers in Svalbard and Jan Mayen (SJG), 4 glaciers in Western Canada and USA (CUG), 9 outlet glaciers in Greenland Periphery (GPG), and 5 outlet glaciers in Antarctic and Subantarctic (ANG)]. N denotes sample size.

Region	Mount	Glacier	LON	LAT	SQ	Distance	Sampling period	Filter	Pore	TDS	Na^+	K^+	Mg^{2+}	Ca^{2+}	N	Source
ASG	ATG	Levyi Aktru Glacier	103.30	50.79	N/A	Subglacial	Jul, 2012	N/A	N/A	N/A	20.0	20.5	153	750	1 [a]	5
	TSG	Urumqi Glacier No.1	86.82	43.10	N/A	0.3	May-Jun, 1996	N/A	N/A	N/A	52.2	45.4	156	689	48	6
					N/A	0.3	Apr-Jun, 1997	GFM	1.00	N/A	51.8	36.4	118	990	38	7
					1.19	0.3	May-Sep, 2006	N/A	0.45	48.3	27.0	23.3	90.0	620	107	8
					0.96	0.3	May-Sep, 2007	N/A	0.45	59.9	27.0	23.3	94.2	730	110	8
					N/A	0.3	May-Sep, 2013	CNM	0.45	39.8 [a]	66.2 [a]	45.7 [a]	157 [a]	936 [a]	248	9
	TSG	Bogeda Glacier	88.27	43.80	N/A	0.5	Jul, 2008	CNM	0.45	15.4	21.4	2.79	45.4	228	72	This study
	TSG	Heigou Glacier	88.36	43.78	N/A	0.1	Aug, 2008	CNM	0.45	17.7	26.6	2.31	41.9	211	50	This study
	TSG	Koxkar Glacier	80.17	41.81	N/A	0.2	Jul-Sep, 2003	N/A	N/A	N/A	139	61.5	217	910	172 [a]	10
					N/A	0.3	Jul, 2008	CNM	0.45	45.6	87.4	47.6	71.4	365	31	This study
	TSG	Donggou Glacier	80.18	41.80	N/A	0.2	Jun, 2008	CNM	0.45	51.4	112	53.4	219	550	36	This study
	TSG	Qingbingtan Glacier	79.91	41.76	N/A	1.1	Aug, 2008	N/A	N/A	N/A	359	131	527	889	30 [b]	11
	QLG	Shuiguanhe Glacier No.2	101.78	37.54	N/A	0.5	Jul, 2007	CNM	0.45	33.0	49.2	13.9	549	468	24	This study
	QLG	Shuiguanhe Glacier No.3	101.78	37.54	N/A	0.3	Jul, 2007	CNM	0.45	38.6	23.2	10.7	512	436	24	This study
	QLG	Shuiguanhe Glacier No.4	101.78	37.54	N/A	0.2	Jul, 2007	CNM	0.45	33.8	19.4	6.44	365	242	24	This study
	QLG	Yanglonghe Glacier No.1	98.59	39.23	N/A	0.1	Aug, 2007	CNM	0.45	41.0	18.3	12.6	215	754	25	This study
	QLG	Yanglonghe Glacier No.5	98.59	39.23	N/A	0.5	Aug, 2007	CNM	0.45	54.9	24.7	13.1	280	698	10	This study
	QLG	Laohugou Glacier	96.55	39.47	N/A	0.1	Aug, 2007	CNM	0.45	34.4	42.8	17.8	231	487	24	This study
	QLG	Qiyi Glacier	97.76	39.23	N/A	2.0	Jun-Jul, 2006	N/A	N/A	N/A	53.9 [c]	22.3 [c]	568 [c]	1023 [c]	26	12
					N/A	0.5	Aug, 2010	CNM	0.45	N/A	98.3 [a]	14.3 [a]	682 [a]	1545 [a]	12	13
					N/A	2.1	Jul-Sep, 2011	N/A	0.45	N/A	127	14.4	334	597	91	14
	KLG	Meikuang Glacier	94.19	35.68	N/A	0.1	Sep-Oct, 2007	CNM	0.45	236	1323	23.3	2605	737	24	This study
	KLG	Yuzhufeng Glacier	94.22	35.63	N/A	0.4	Oct, 2007	CNM	0.45	177	872	29.3	1702	802	24	This study

TGG	Dongkemadi Glacier	92.07	33.07	N/A	0.3	Oct, 2007	CNM	0.45	46.5	70.6	38.1	103	674	23	This study
				0.98	0.5	Jun-Sep, 2013	CNM	0.45	N/A	37.7[d]	12.8[d]	84.3[d]	568[d]	48	15
PAG	Gongger Glacier No.1	75.27	38.48	N/A	0.4	Jun, 2008	CNM	0.45	53.4	42.9	43.7	88.7	561	24	This study
PAG	Gongger Glacier No.2	75.05	38.67	N/A	0.3	Jun, 2008	CNM	0.45	70.4	55.5	81.6	119	1069	25	This study
PAG	Gongger Glacier No.3	75.19	38.51	N/A	0.5	Jun, 2008	CNM	0.45	77.5	140	177	90.3	817	26	This study
PAG	Kartamak Glacier	75.07	38.28	N/A	<1.5	Aug, 2003	N/A	N/A	N/A	8.19[d]	8.74[d]	14.5[d]	311[d]	13	16
HDG	Hailuogou Glacier	101.98	29.57	N/A	0.3	Apr, 2008	CNM	0.45	47.9	41.2	118	68.4	605	51	This study
				N/A	0.3	May-Sep, 2008	CNM	0.45	29.8[a]	17.9[a]	66.7[a]	51.2[a]	437[a]	144	17
				N/A	1.1	Apr-Nov, 2013	CNM	0.45	51.2[a]	32.0[a]	84.1[a]	58.0[a]	535[a]	379	17
HDG	Hailuogou Glacier No.2	101.97	29.55	N/A	0.5	Apr, 2008	CNM	0.45	48.2	22.7	97.1	68.5	734	49	This study
HMG	Rongbulk Glacier	86.86	28.08	N/A	N/A	Jun-Oct, 1998	N/A	N/A	N/A	43.9	63.3	75.0	403	117	18
				N/A	0.5	Jun, 2008	CNM	0.45	46.1	33.4	23.6	101	602	25	This study
HMG	B&B Glacier	74.83	36.08	N/A	0.6	May-Jun, 2015	CEM	0.45	136	30.4	66.7	425	1190	8	19
HMG	Bara Shigri Glacier	77.67	32.17	N/A	3.0	Aug-Sep, 2011	MCM	0.45	33.8	28.4	28.8	60.2	274	19	20
				N/A	3.0	Jul-Sep, 2012	MCM	0.45	25.8	21.3	38.1	73.7	169	20	21
				N/A	3.0	Aug-Sep, 2013	MCM	0.45	33.7	27.4	42.2	89.7	344	15	21
HMG	Batal Glacier	77.58	32.34	N/A	1.0	Aug-Sep, 2014	MCM	0.45	81.7	34.0	29.5	224	838	24	22
				N/A	1.0	Jun-Sep, 2015/2017	MCM	0.45	48.5	50.2	30.0	101	559	61	23
HMG	Batura Glacier	74.70	37.30	1.60	0.5	Jun-Jul, 1999	CNM	0.45	N/A	56.5[e]	46.2[e]	233[e]	600[e]	15	24
HMG	Bilare Banga Glacier	78.38	31.30	N/A	<3.0	Jun-Jul, 2017[a]	N/A	0.45	N/A	163	52.2	199	288	12[a]	25
HMG	Chaturangi Glacier	79.18	30.90	N/A	<1.0	Aug-Sep, 2008	N/A	0.45	84.9	105	85.8	405	490	14	26
HMG	Chhota Shigri Glacier	77.52	32.23	N/A	1.5	Jul-Aug, 1987	N/A	0.45	N/A	30.9	25.1	11.7	117	31	27
				N/A	2.0	Sep-Oct, 2003	MCM	0.45	58.4	94.0	60.0	201	335	40	28
				N/A	2.0	Sep-Oct, 2004	MCM	0.45	62.8	105	85.0	224	325	40	28
				N/A	2.0	Sep-Oct, 2005	MCM	0.45	79.4	116	88.0	281	527	40	28
				N/A	2.0	Sep-Oct, 2006	MCM	0.45	39.6	57.0	44.0	125	281	40	28
				N/A	2.0	Sep-Oct, 2007	MCM	0.45	35.3	52.0	39.0	115	225	40	28
				N/A	2.0	Aug-Oct, 2008	N/A	0.45	38.3	58.3	51.7	118	128	63	29
				N/A	2.0	Aug-Oct, 2009	N/A	0.45	27.0	38.8	28.9	98.8	104	118	29
				N/A	2.0	May-Oct, 2010	N/A	0.45	27.9	37.9	30.8	74.2	150	164	30
HMG	Dokriani Glacier	78.43	33.57	N/A	0.6	May-Oct, 1994	N/A	0.45	N/A	29.0	69.3	64.6	544	28	31
HMG	Dudu Glacier	78.80	30.98	N/A	2.5	Jun-Oct, 1997	N/A	0.45	N/A	42.7	24.6	6.50	91.1	24	32
HMG	Gangotri Glacier	79.17	30.82	N/A	0.5	Jun-Sep, 2003-2004	N/A	0.45	N/A	48.3	43.8	1330	620	48	33
				N/A	1.5	Jul-Sep, 2007	N/A	0.45	51.0	75.0	83.0	197	206	52	34

					N/A	2.0	May-Sep, 2008	N/A	0.45	51.5 [f]	48.4 [f]	54.3 [f]	220 [f]	259 [f]	142 [a]	35
					N/A	Snout	May-Oct, 2014-2016	N/A	0.45	56.7	42.4	44.1	162	518	102 [a]	36
	HMG	Gulmit Glacier	74.77	36.42	N/A	0.4	May-Jun, 2015	CEM	0.45	37.2	21.7	82.1	25.0	335	8	19
	HMG	Hinarchi Glacier	74.58	36.12	N/A	0.2	May-Jun, 2015	CEM	0.45	109	30.4	56.4	225	1010	7	19
	HMG	Khimsung Glacier	85.63	28.17	N/A	<5.0	Apr-Jul, 2014	N/A	N/A	10.6	29.6	26.4	91.7	283	2 [a]	37
	HMG	Lirung Glacier	85.58	28.17	N/A	<5.0	Apr-Jul, 2014	N/A	N/A	23.0	54.3	40.5	90.0	433	2 [a]	37
	HMG	Manimahesh Glacier	76.67	32.38	N/A	<2.0	Jun-Jul, 2014	N/A	0.45	18.0	9.00	12.0	18.0	165	21	38
	HMG	Passu Glacier	74.75	36.48	N/A	0.8	May-Jun, 2015	CEM	0.45	35.9	26.1	51.3	50.0	245	8	19
	HMG	Patsio Glacier	77.35	32.79	N/A	1.5	Sep, 2010	N/A	0.45	94.9	34.8	38.1	275	890	24	39
					N/A	1.5	Sep, 2011	N/A	0.45	95.5	42.2	52.6	320	982	28	39
					N/A	1.5	Sep, 2012	N/A	0.45	105	42.5	46.4	305	1035	20	39
	HMG	Sutri Dhaka Glacier	77.56	32.38	N/A	0.2	Jul-Aug, 2015	N/A	0.45	N/A	5.00	14.7	43.6	242	59	40
	HMG	Naradu Glacier	78.40	31.28	N/A	<5.0	Jun-Jul, 2016 [a]	MCM	0.45	N/A	167	68.0	180	260	21	41
					N/A	<5.0	Jun-Jul, 2017 [a]	MCM	0.45	N/A	179	52.2	193	265	26	41
ALG	N/A	Bench Glacier	-148.50	62.18	N/A	Snout	Jun, 1996	N/A	0.45	N/A	25.0	61.0	36.0	550	>5	42
	N/A	Kennicott Glacier	-142.96	61.55	N/A	0.50	Jul-Sep, 1999		0.45	N/A	63.0	14.0	160	932	60	43
					N/A	0.50	Jun-Sep, 2000		0.45	N/A	87.0	17.0	176	1002	93	43
ACG	N/A	John Evans Glacier	-74.00	79.67	N/A	Subglacial	Jun-Aug, 1994	CNM	0.45	N/A	54.0	12.1	175	1701	38	44
					N/A	Subglacial	Jun-Jul, 1996	CNM	0.45	N/A	45.0	9.51	176	1610	8	44
CEG	N/A	Damma Glacier	8.47	46.75	N/A	<0.1	May-Oct, 2008	NYF	0.20	N/A	9.12	12.0	6.57	31.1	12	45
	N/A	Glacier de Tsanfleuron	7.22	46.32	N/A	N/A	Jul-Sep, 1974-1977/1990-1991	N/A	0.45	N/A	12.1 [g]	10.4 [g]	247 [g]	878 [g]	44	46
	N/A	Gornergletscher	7.80	45.97	N/A	0.25	Jul-Aug, 1974	OXF	0.45	N/A	19.1	13.1	77.5	216	69	47
					N/A	0.25	Jul-Sep, 1975	OXF	0.45	N/A	12.2	19.0	76.7	187	59	47
					N/A	0.25	Jul-Aug, 1976	OXF	0.45	N/A	13.5	15.1	88.3	253	41	47
	N/A	Haut Glacier d'Arolla	8.38	46.45	N/A	0.20	Jun-Aug, 1999	CNM	0.45	N/A	16.1 [h]	9.49 [h]	33.3 [h]	330 [h]	132	48
	N/A	Marmolada Glacier	11.85	46.82	N/A	<0.1	Aug, 1990	N/A	0.45	N/A	N/A	N/A	46.4 [i]	713 [i]	6	46
ICG	N/A	Fjallsjökull Glacier	-16.36	64.08	N/A	Snout	Jul/Aug, 1976 [a]	N/A	N/A	N/A	83.7	5.53	51.2	243	18	49
	N/A	Vatnajökull Glacier	-16.80	64.42	N/A	<3.0	Jul, 2011	CAM	0.20	113 [j]	278 [j]	9.64 [j]	176 [j]	892 [j]	1	50
LLG	N/A	Rio Quilcay	-76.44	-10.16	N/A	<5.0	Jul, 2008	N/A	0.40	N/A	47.6 [k]	14.9 [k]	495 [k]	1017 [k]	16	51
SCG	N/A	Austre Okstindbreen	14.12	66.02	N/A	N/A	Jul, 1983	N/A	0.45	N/A	54.8	7.95	20.8	17.5	N/A	52
					N/A	N/A	Jul, 1984	N/A	0.45	N/A	29.1	5.90	8.33	11.5	N/A	52
					N/A	N/A	Jul, 1985	N/A	0.45	N/A	23.0	14.9	27.5	165	N/A	52

					N/A	N/A	Jul, 1986	N/A	0.45	N/A	29.1	28.7	41.7	283	N/A	52
					N/A	N/A	Jul, 1987	N/A	0.45	N/A	34.8	5.90	15.0	28.0	N/A	52
					N/A	N/A	Jul, 1988	N/A	0.45	N/A	14.8	4.36	14.2	39.5	N/A	52
					N/A	N/A	Jul, 1989	N/A	0.45	N/A	137	8.97	40.8	33.5	N/A	52
					N/A	N/A	Jul, 1990	N/A	0.45	N/A	75.2	6.92	23.3	33.0	N/A	52
SJG	N/A	Longyearbreen	15.50	78.18	N/A	<0.1	Aug, 2004	CNM	0.45	N/A	541[a]	21.0[a]	905[a]	814[a]	183	53
	N/A	Rieperbreen	16.20	78.13	N/A	0.10	Jun-Sep, 2007	CNM	0.45	N/A	865	30.3	524	1020	16	54
	N/A	Scott Turnerbreen	15.95	78.10	N/A	<0.5	Jun-Jul, 1993	CNM	0.45	N/A	430	12.0	210	220	72	55
	N/A	Werenskioldbreen	15.25	77.08	N/A	<2.0	May-Sep, 2011	NYF	0.45	N/A	190	17.0	263	734	183	56
CUG	N/A	Berendon Glacier	-129.45	53.48	N/A	Snout	Jul-Aug, 1975	N/A	0.45	N/A	4.35[l]	2.77[l]	10.4[l]	428[l]	2	57
	N/A	Eliot Glacier	-121.60	45.36	N/A	<1.0	Jul, 2005	N/A	N/A	N/A	39.0	5.30	42.0	86.0	10	58
	N/A	South Cascade Glacier	-121.06	48.36	N/A	Snout	Aug, 1992	CAM	0.22	N/A	9.57[m]	23.2[m]	10.5[m]	71.4[m]	N/A	59
	N/A	Saskatchewan Glacier	-118.45	53.00	N/A	<0.5	Sep, 1991	N/A	0.45	N/A	17.9[i]	N/A[i]	305[i]	637[i]	12	46
GPG	N/A	Glacier ‘G’	-38.46	65.71	N/A	<0.03	Aug, 2013	PFM	0.20	N/A	45.4	10.0	20.2	57.8	5	60
	N/A	Isunnguata Sermia Glacier	-50.10	67.20	N/A	Snout	Jul, 2013	NYF	0.10	N/A	32.7	22.2	10.3	104	45	61
	N/A	Kangaarsarsuup Glacier	-49.95	64.10	N/A	<0.03	Aug, 2013	PFM	0.20	N/A	25.6	20.5	14.0	71.5	3	60
	N/A	Leverett Glacier	-52.20	67.06	N/A	1.00	Jul-Aug, 2009	NYF	0.20	N/A	41.0[n]	27.2[n]	17.6[n]	70.9[n]	74	62
					N/A	<3.0	May-Sep, 2009	CNM	0.45	N/A	33.2[a]	21.2[a]	19.4[a]	79.1[a]	99	63
					N/A	<3.0	Apr-Aug, 2010	CNM	0.45	N/A	37.9[a]	18.7[a]	12.7[a]	74.8[a]	143	63
					3.70	<3.0	May-Aug, 2012	CNM	0.45	N/A	48.0[a]	27.9[a]	21.0[a]	101[a]	73	63
					N/A	<3.0	May-Sep, 2015	GXF	0.45	N/A	53.1[a]	28.5[a]	39.0[a]	155[a]	129	63
	N/A	Kiattuut Sermiat Glacier	-45.33	61.21	6.10	<3.0	Apr-Aug, 2013	CNM	0.45	N/A	30.0[a]	20.8[a]	37.6[a]	284[a]	104	63
	N/A	Qooqqup Glacier	-45.33	61.21	N/A	<0.03	Jul, 2013	PFM	0.20	N/A	47.3	17.3	38.9	181	4	60
	N/A	Russell Glacier	-50.07	67.13	N/A	<4.0	Jul, 2006	N/A	0.20	7.99[o]	9.83[o]	11.0[o]	18.0[o]	55.9[o]	2	64
					N/A	<0.03	Aug, 2013	PFM	0.20	N/A	3.38	3.60	6.65	21.1	4	60
					N/A	0.10	Jun-Aug, 2014	N/A	0.45	N/A	6.50[p]	4.76[p]	13.7[p]	35.4[p]	7	65
					N/A	0.10	Jun-Aug, 2015	N/A	0.45	N/A	12.4[p]	7.03[p]	23.0[p]	63.0[p]	12	65
	N/A	Imersuaq Glacier	-49.90	66.17	N/A	Snout	Jul, 2000	CNM	0.45	N/A	153[a]	61.8[a]	173[a]	147[a]	63	66
	N/A	Glacier “N”	-50.27	68.04	2.50	Snout	May-Jul, 2008	CAM	0.22	N/A	24.5[q]	22.0[q]	41.0[q]	54.2[q]	40	67
ANG	N/A	Clark Glacier	162.37	-77.42	N/A	<5.0	Jan, 2010	N/A	0.2	N/A	180	49.9	169	301	6	68
	N/A	Denton Glacier	162.58	-77.48	N/A	N/A	N/A	N/A	N/A	N/A	551	78.2	286	800	1	68
	N/A	Goldman Glacier	162.85	-77.70	N/A	N/A	N/A	N/A	N/A	N/A	266	40.9	200	959	5	68

N/A	Howard Glacier	163.08	-77.68	N/A	<5.0	Jan, 2010	N/A	0.2	N/A	216	26.7	114	776	4	68
N/A	Moa Glacier	162.78	-77.72	N/A	N/A	N/A	N/A	N/A	N/A	84.4	26.9	82.7	224	1	68

Note. N/A denotes no available data. CNM, MCM, CAM, NYF, OXF, GXF, PFM, GFM and CEM denote cellulose nitrate membrane, millipore cellulose membrane, cellulose acetate membrane, nylon filter, oxoid filter, GD/XP PES filter, polyvinylidene fluoride membrane, glass fibre membrane and cellulose ester membrane respectively.

[a] Value and/or relevant information supplied by the first author;
[b] Value from the counting of data points in figure 2;
[c] Value from the sampling site TB;
[d] Value from the sampling site S1;
[e] Value denotes the discharge-weighted mean concentration;
[f] Value denotes the mean concentration during the pre-monsoon, monsoon and post-monsoon seasons;
[g] Value from the main meltstream (Central Tsanfleuron), streams (North Tsanfleuron), and streams and pools (South Tsanfleuron);
[h] Value denotes the median concentration;
[i] Value from the streams and pools;
[j] Value from the sampling site Sveðja;
[k] Value from the sampling sites 1 – 16;
[l] Value from the samples R10 and R11;
[m] Value from the stream 2 and stream 4;
[n] Value from the samples 0705am to 0801am;
[o] Value from the sampling sites GR1 and GR9;
[p] Value from the subglacial samples;
[q] Value from all samples apart from sample G12.

Supplementary Table 4: Mean concentrations (Con; ±std; μeq L^{-1}) and the percentages (Per; ±std; %) of major cations as well as total dissolved solids (TDS; ±std; mg L^{-1}) for 19 glaciers in seven mountain ranges (Mount) within Asia generated in this study (Supplementary Tables 1 and 3). N denotes sample size.

Mount	Glacier	TDS		Na^+		K^+		Mg^{2+}		Ca^{2+}		Na^+		K^+		Mg^{2+}		Ca^{2+}		
		Con	Std	Con	Std	Con	Std	Con	Std	Con	Std	Per	Std	Per	Std	Per	Std	Per	Std	N
TSG	KOG	45.6	16.1	87.4	38.6	47.6	15.3	71.4	34.6	365	89.3	15.3	21.7	8.33	8.59	12.5	19.5	63.9	50.2	31
	DGG	51.4	11.9	112	34.4	53.4	12.0	219	53.0	550	91.2	12.0	18.1	5.72	6.30	23.5	27.8	58.8	47.8	36
	BGG	15.4	2.18	21.4	4.92	2.79	0.83	45.4	9.83	228	31.3	7.19	10.5	0.94	1.78	15.3	21.0	76.6	66.8	72
	HGG	17.7	4.61	26.6	7.57	2.31	0.62	41.9	12.6	211	70.2	9.44	8.32	0.82	0.68	14.9	13.8	74.9	77.2	50
QLG	SG2	33.0	11.9	49.2	20.6	13.9	5.76	549	198	468	92.2	4.55	6.52	1.28	1.82	50.8	62.5	43.3	29.1	24
	SG3	38.6	10.6	23.2	9.49	10.7	4.76	512	223	436	137	2.36	2.54	1.09	1.27	52.1	59.6	44.4	36.6	24
	SG4	33.8	8.50	19.4	6.24	6.44	1.46	365	125	242	77.3	3.06	2.96	1.02	0.69	57.7	59.6	38.3	36.8	24
	YG1	41.0	10.0	18.3	6.63	12.6	4.00	215	90.1	754	199	1.83	2.21	1.26	1.33	21.5	30.0	75.4	66.4	25
	YG5	54.9	10.2	24.7	5.29	13.1	2.83	280	75.1	698	125	2.44	2.54	1.29	1.36	27.5	36.0	68.7	60.1	10
	LHG	34.4	9.19	42.8	20.3	17.8	5.52	231	73.2	487	182	5.49	7.23	2.28	1.97	29.7	26.1	62.5	64.7	24
KLG	MKG	236	67.8	1323	378	23.3	6.63	2605	763	737	188	28.2	28.3	0.50	0.50	55.6	57.1	15.7	14.1	24
	YZG	177	50.3	872	267	29.3	9.78	1702	576	802	183	25.6	25.8	0.86	0.95	50.0	55.6	23.6	17.7	24
TGG	DKG	53.4	1.06	70.6	6.64	38.1	4.78	103	9.59	674	57.2	7.97	8.48	4.30	6.11	11.6	12.3	76.1	73.2	23
PAG	GG1	53.4	6.83	42.9	8.63	43.7	6.41	88.7	16.1	561	123	5.82	5.61	5.93	4.16	12.1	10.4	76.2	79.8	24
	GG2	70.4	18.8	55.5	22.4	81.6	22.0	119	49.3	1069	443	4.19	4.18	6.16	4.10	8.97	9.19	80.7	82.5	25
	GG3	77.5	7.20	140	12.2	177	14.2	90.3	10.0	817	112	11.4	8.19	14.4	9.58	7.37	6.71	66.7	75.5	26
HDG	HLG	47.9	5.66	41.2	8.11	118	11.4	68.4	10.7	605	124	4.95	5.25	14.2	7.39	8.22	6.91	72.7	80.4	51
	HG2	48.2	9.00	22.7	5.53	97.1	17.1	68.5	13.3	734	109	2.47	3.82	10.5	11.8	7.43	9.20	79.6	75.2	49
HMG	RBG	46.1	6.29	33.4	5.51	23.6	3.61	101	21.3	602	72.7	4.40	5.35	3.11	3.50	13.3	20.7	79.2	70.5	25
	Average	61.5	54.1	159	341	42.7	46.0	393	655	581	225	13.5	26.9	3.63	3.63	33.4	51.7	49.4	17.8	591

Supplementary Table 5: Mean concentrations (Con; ±std; μeq L^{-1}) and the percentages (Per; ±std; %) of major cations as well as total dissolved solid (TDS; ±std; mg L^{-1}) for 43 glaciers in eight mountain ranges within Asia (ATG, TSG, QLG, KLG, TGG, PAG, HDG, and HMG) and for 77 glaciers in eleven glacial regions worldwide (ASG, ALG, ACG, CEG, ICG, LLG, SCG, SJG, CUG, GPG, and ANG; Supplementary Tables 1 and 3). Note that ATG denotes the Altai mountain range in Asia, and N denotes sample size.

		TDS		Na^+		K^+		Mg^{2+}		Ca^{2+}		Na^+		K^+		Mg^{2+}		Ca^{2+}		
		Con	Std	Con	Std	Con	Std	Con	Std	Con	Std	Per	Std	Per	Std	Per	Std	Per	Std	N
Mountain range	ATG	N/A	N/A	20.0	N/A	20.5	N/A	153	N/A	750	N/A	2.12	N/A	2.18	N/A	16.2	N/A	79.5	N/A	1
	TSG	39.7	17.0	88.2	97.7	42.9	35.0	158	137	647	282	9.42	17.7	4.59	6.35	16.9	24.8	69.1	51.1	942
	QLG	39.3	8.27	50.7	38.0	13.9	4.39	415	167	694	389	4.32	6.35	1.19	0.73	35.3	27.9	59.2	65.0	260
	KLG	206	41.9	1097	318	26.3	4.20	2153	639	770	46.0	27.1	31.6	0.65	0.42	53.2	63.4	19.0	4.57	48
	TGG	46.5	N/A	54.1	23.2	25.4	17.8	93.5	13.1	621	75.1	6.82	18.0	3.20	13.8	11.8	10.1	78.2	58.1	71
	PAG	67.1	12.4	61.7	56.0	77.7	72.5	78.1	44.6	690	327	6.80	11.2	8.57	14.5	8.61	8.92	76.0	65.4	88
	HDG	44.3	9.81	28.5	10.3	91.5	21.6	61.5	8.47	578	125	3.75	6.26	12.0	13.1	8.10	5.13	76.1	75.5	623
	HMG	55.2	31.5	55.5	42.0	47.9	20.3	185	218	430	290	7.73	7.37	6.66	3.56	25.8	38.2	59.8	50.8	1528
	Average	56.8	40.2	87.6	185	76.8	53.3	130	203	469	266	11.5	26.2	10.1	7.53	17.0	28.8	61.4	37.6	3561
Glacial region	ASG	56.8	40.2	87.6	185	45.3	31.4	249	390	539	306	9.51	20.3	4.92	3.44	27.1	42.7	58.5	33.5	3561
	ALG	N/A	N/A	58.3	31.3	30.7	26.3	124	76.6	828	243	5.60	8.28	2.95	6.97	11.9	20.3	79.5	64.4	158
	ACG	N/A	N/A	49.5	6.36	10.8	1.81	176	0.71	1656	64.3	2.62	8.69	0.57	2.48	9.28	0.97	87.5	87.9	46
	CEG	N/A	N/A	13.7	3.50	13.2	3.47	82.2	78.0	372	307	2.84	0.89	2.74	0.89	17.1	19.9	77.4	78.3	363
	ICG	113	N/A	181	137	7.59	2.90	114	88.3	568	459	20.8	20.0	0.87	0.42	13.1	12.8	65.3	66.7	19
	LLG	N/A	N/A	47.6	N/A	14.9	N/A	495	N/A	1017	N/A	3.02	N/A	0.95	N/A	31.4	N/A	64.6	N/A	16
	SCG	N/A	N/A	49.7	40.0	10.4	8.04	24.0	12.2	76.3	96.6	31.0	25.5	6.52	5.13	14.9	7.77	47.6	61.6	N/A
	SJG	N/A	N/A	507	280	20.1	7.73	476	318	697	340	29.8	29.6	1.18	0.82	28.0	33.6	41.0	36.0	454
	CUG	N/A	N/A	17.7	15.3	10.4	11.1	92.0	143	305	276	4.16	3.43	2.45	2.50	21.6	32.1	71.8	62.0	>24
	GPG	7.99	N/A	37.7	34.4	20.3	13.7	31.6	39.4	97.2	66.2	20.2	22.4	10.9	8.90	16.9	25.6	52.0	43.1	807
	ANG	N/A	N/A	259	176	44.5	21.3	170	79.3	612	328	23.9	29.1	4.10	3.52	15.7	13.1	56.4	54.2	17
	Average	57.0	40.8	93.3	177	34.6	29.0	191	321	472	366	11.8	19.8	4.37	3.25	24.2	35.9	59.7	41.0	>5465

Note. N/A denotes no available data.

Supplementary Table 6: Total dissolved solids (TDS; mg L^{-1}) and the molar ratios of cations for 77 glaciers in eleven glacial regions worldwide (Supplementary Table 3). These glaciers include 43 glaciers (including the 19 Asian glaciers generated in this study) in Asia (ASG), 2 glaciers in Alaska (ALG), 1 glacier in Arctic Canada (ACG), 5 glaciers in Central Europe (CEG), 2 glaciers in Iceland (ICG), 1 glacier in Low Latitudes (LLG), 1 glacier in Scandinavia (SCG), 4 glaciers in Svalbard and Jan Mayen (SJG), 4 glaciers in Western Canada and USA (CUG), 9 outlet galciers in Greenland Periphery (GPG), and 5 outlet glaciers in Antarctic and Subantarctic (ANG).

Region	Glacier	Sampling period	TDS	Na/(Na+Ca)	Ca/Na	Mg/Na
ASG	Levyi Aktru Glacier	Jul, 2012	N/A	0.05	18.8	3.81
	Glacier No.1	May-Jun, 1996	N/A	0.13	6.60	1.49
		Apr-Jun, 1997	N/A	0.09	9.55	1.14
		May-Sep, 2006	48.3	0.08	11.5	1.67
		May-Sep, 2007	59.9	0.07	13.5	1.75
		May-Sep, 2013	39.8	0.12	7.07	1.18
	Bogeda Glacier (BGG)	Jul, 2008	15.4	0.16	5.33	1.06
	Heigou Glacier (HGG)	Aug, 2008	17.7	0.20	3.97	0.79
	Koxkar Glacier (KOG)	Jul-Sep, 2003	N/A	0.23	3.27	0.78
		Jul, 2008	45.6	0.32	2.09	0.41
	Donggou Glacier (DGG)	Jun, 2008	51.4	0.29	2.45	0.98
	Qingbingtan Glacier	Aug, 2008	N/A	0.45	1.24	0.73
	Shuiguanhe Glacier No.2 (SG2)	Jul, 2007	33.0	0.17	4.76	5.58
	Shuiguanhe Glacier No.3 (SG3)	Jul, 2007	38.6	0.10	9.41	11.0
	Shuiguanhe Glacier No.4 (SG4)	Jul, 2007	33.8	0.14	6.25	9.42
	Yanglonghe Glacier No.1 (YG1)	Aug, 2007	41.0	0.05	20.6	5.86
	Yanglonghe Glacier No.5 (YG5)	Aug, 2007	54.9	0.07	14.1	5.65
	Laohugou Glacier (LHG)	Aug, 2007	34.4	0.15	5.69	2.70
	Qiyi Glacier	Jun-Jul, 2006	N/A	0.10	9.49	5.26
		Aug, 2010	N/A	0.11	7.86	3.47
		Jul-Sep, 2011	N/A	0.30	2.36	1.32
	Meikuang Glacier (MKG)	Sep-Oct, 2007	236	0.78	0.28	0.98
	Yuzhufeng Glacier (YZG)	Oct, 2007	177	0.69	0.46	0.98
	Dongkemadi Glacier (DKG)	Oct, 2007	46.5	0.17	4.78	0.73
		Jun-Sep, 2013	N/A	0.12	7.53	1.12
	Gongger Glacier No.1 (GG1)	Jun, 2008	53.4	0.13	6.54	1.04
	Gongger Glacier No.2 (GG2)	Jun, 2008	70.4	0.09	9.63	1.07

	Gongger Glacier No.3 (GG3)	Jun, 2008	77.5	0.26	2.91	0.32
	Kartamak Glacier	Aug, 2003	N/A	0.05	19.0	0.88
	Hailuogou Glacier (HLG)	Apr, 2008	47.9	0.12	7.33	0.83
		May-Sep, 2008	29.8	0.08	12.2	1.43
		Apr-Nov, 2013	51.2	0.11	8.35	0.90
	Hailuogou Glacier No.2 (HG2)	Apr, 2008	48.2	0.06	16.1	1.51
	Rongbulk Glacier (RBG)	Jun-Oct, 1998	N/A	0.18	4.59	0.85
		Jun, 2008	46.1	0.10	9.01	1.51
	B&B Glacier	May-Jun, 2015	136	0.05	19.6	6.98
	Bara Shigri Glacier	Aug-Sep, 2011	33.8	0.17	4.83	1.06
		Jul and Sep, 2012	25.8	0.20	3.97	1.73
		Aug-Sep, 2013	33.7	0.14	6.28	1.64
	Batal Glacier	Aug-Sep, 2014	81.7	0.08	12.3	3.29
		Jun-Jul and Sep, 2015/2017	48.5	0.15	5.57	1.01
	Batura Glacier	Jun-Jul, 1999	N/A	0.16	5.31	2.06
	Bilare Banga Glacier	Melt season, 2017	N/A	0.53	0.89	0.61
	Chaturangi Glacier	Aug-Sep, 2008	84.9	0.30	2.33	1.93
	Chhota Shigri Glacier	Jul-Aug, 1987	N/A	0.35	1.89	0.19
		Sep-Oct, 2003	58.4	0.36	1.78	1.07
		Sep-Oct, 2004	62.8	0.39	1.55	1.07
		Sep-Oct, 2005	79.5	0.31	2.27	1.21
		Sep-Oct, 2006	39.6	0.29	2.46	1.10
		Sep-Oct, 2007	35.3	0.32	2.16	1.11
		Aug-Oct, 2008	38.3	0.48	1.10	1.01
		Aug-Oct, 2009	27.0	0.43	1.34	1.27
		May-Oct, 2010	27.9	0.34	1.98	0.98
	Dokriani Glacier	May-Oct, 1994	N/A	0.10	9.37	1.11
	Dudu Glacier	Jun and Oct, 1997	N/A	0.48	1.07	0.08
	Gangotri Glacier	Jun-Sep, 2003-2004	N/A	0.13	6.42	13.8
		Jul-Sep, 2007	51.0	0.42	1.37	1.31
		May-Sep, 2008	51.5	0.27	2.67	2.27
		May-Oct, 2014-2016	56.7	0.14	6.11	1.91
	Gulmit Glacier	May-Jun, 2015	37.2	0.11	7.71	0.58
	Hinarchi Glacier	May-Jun, 2015	109	0.06	16.6	3.70
	Khimsung Glacier	Apr and Jun-Jul, 2014	10.6	0.17	4.79	1.55

	Lirung Glacier	Apr and Jun-Jul, 2014	23.0	0.20	3.98	0.83
	Manimahesh Glacier	Jun-Jul, 2014	18.0	0.10	9.17	1.00
	Passu Glacier	May-Jun, 2015	35.9	0.18	4.70	0.96
	Patsio Glacier	Sep, 2010	94.9	0.07	12.8	3.95
		Sep, 2011	95.5	0.08	11.6	3.79
		Sep, 2012	105	0.08	12.2	3.59
	Sutri Dhaka Glacier	Jul-Aug, 2015	N/A	0.04	24.2	4.36
	Naradu Glacier	Summer, 2016	N/A	0.56	0.78	0.54
		Summer, 2017	N/A	0.57	0.74	0.54
ALG	Bench Glacier	Jun, 1996	N/A	0.08	11.0	0.72
	Kennicott Glacier	Jul-Sep, 1999	N/A	0.12	7.40	1.27
		Jun-Sep, 2000	N/A	0.15	5.76	1.01
ACG	John Evans Glacier	Jun-Aug, 1994	N/A	0.06	15.8	1.62
		Jun-Jul, 1996	N/A	0.05	17.9	1.96
CEG	Damma Glacier	May-Oct, 2008	N/A	0.37	1.70	0.36
	Glacier de Tsanfleuron	Jul-Sep, 1974-1977/1990-1991	N/A	0.03	36.3	10.2
	Gornergletscher	Jul-Aug, 1974	N/A	0.15	5.65	2.03
		Jul-Sep, 1975	N/A	0.12	7.66	3.15
		Jul-Aug, 1976	N/A	0.10	9.37	3.28
	Haut Glacier d'Arolla	Jun-Aug, 1999	N/A	0.09	10.3	1.04
	Marmolada Glacier	Aug, 1990	N/A	N/A	N/A	N/A
ICG	Fjallsjökull Glacier	Jul/Aug, 1976	N/A	0.41	1.45	0.31
	Vatnajökull Glacier	Jul, 2011	113	0.38	1.60	0.32
	Rio Quilcay	Jul, 2008	N/A	0.09	10.7	5.20
SCG	Austre Okstindbreen	Jul, 1983	N/A	0.86	0.16	0.19
		Jul, 1984	N/A	0.84	0.20	0.14
		Jul, 1985	N/A	0.22	3.57	0.60
		Jul, 1986	N/A	0.17	4.85	0.72
		Jul, 1987	N/A	0.71	0.40	0.22
		Jul, 1988	N/A	0.43	1.34	0.48
		Jul, 1989	N/A	0.89	0.12	0.15
		Jul, 1990	N/A	0.82	0.22	0.16
SJG	Longyearbreen	Aug, 2004	N/A	0.57	0.75	0.84
	Rieperbreen	Jun-Sep, 2007	N/A	0.63	0.59	0.30
	Scott Turnerbreen	Jun-Jul, 1993	N/A	0.80	0.26	0.24

	Werenskioldbreen	May-Sep, 2011	N/A	0.34	1.93	0.69
CUG	Berendon Glacier	Jul-Aug, 1975	N/A	0.02	49.2	1.20
	Eliot Glacier	Jul, 2005	N/A	0.48	1.10	0.54
	South Cascade Glacier	Aug, 1992	N/A	0.21	3.73	0.55
	Saskatchewan Glacier	Sep, 1991	N/A	0.05	17.8	8.51
GPG	Glacier “G”	Aug, 2013	N/A	0.61	0.64	0.22
	Isunnguata Sermia Glacier	Jul, 2013	N/A	0.39	1.58	0.16
	Kangaarsarsuup Glacier	Aug, 2013	N/A	0.42	1.40	0.27
	Leverett Glacier	Jul-Aug, 2009	N/A	0.54	0.86	0.21
		May-Sep, 2009	N/A	0.46	1.19	0.29
		Apr-Aug, 2010	N/A	0.50	0.99	0.17
		May-Aug, 2012	N/A	0.49	1.05	0.22
		May-Sep, 2015	N/A	0.41	1.46	0.37
	Kiattuut Sermiat Glacier	Apr-Aug, 2013	N/A	0.17	4.72	0.63
	Qooqqup Glacier	Jul, 2013	N/A	0.34	1.91	0.41
	Russell Glacier	Jul, 2006	7.99	0.26	2.84	0.92
		Aug, 2013	N/A	0.24	3.12	0.99
		Jun-Aug, 2014	N/A	0.27	2.73	1.05
		Jun-Aug, 2015	N/A	0.28	2.55	0.93
	Imersuaq Glacier	Jul, 2000	N/A	0.67	0.48	0.57
	Glacier “N”	May-Jul, 2008	N/A	0.48	1.10	0.84
ANG	Clark Glacier	Jan, 2010	N/A	0.54	0.84	0.47
	Denton Glacier	Jan, 2010	N/A	0.58	0.73	0.26
	Goldman Glacier	Jan, 2010	N/A	0.36	1.80	0.38
	Howard Glacier	Jan, 2010	N/A	0.36	1.80	0.26
	Moa Glacier	Jan, 2010	N/A	0.43	1.33	0.49

Note. N/A denotes no available data.

Supplementary Table 7: Mineral composition (%) of glacial deposits for Koxkar Glacier (KOG; *n* = 10) in the Tianshan generated in this study in comparison to four other Asian glaciers. These glaciers include Urumqi Glacier No.1 (*n* = 5) in the Tianshan, Qiyi Glacier (*n* = 8) in the Qilian, Dongkemadi Glacier (DKG; *n* = 8) in the Tanggula, and Hailuogou Glacier (HLG; *n* = 5) in the Hengduan mountain ranges.

Glacier	Sample	Montmorillonite	Illite	Gypsum	Kaolinite	Chlorite	Quartz	Potash feldspar	Plagioclase	Calcite	Dolomite	Siderite	Hematite	Pyrite	Source
Koxkar Glacier	KOG01	N/D	4.7	N/D	N/D	1.1	29.7	34.9	27.4	0.7	1.5	N/D	N/D	N/D	This study
	KOG02	N/D	5.1	N/D	N/D	N/D	23.6	45.3	23.8	0.8	0.7	0.7	N/D	N/D	This study
	KOG03	N/D	5.6	0.1	1.3	N/D	25.9	44.0	21.5	0.7	0.5	0.4	N/D	N/D	This study
	KOG04	N/D	1.2	N/D	N/D	N/D	37.3	36.0	23.4	1.0	0.5	0.3	N/D	0.3	This study
	KOG05	N/D	N/D	N/D	1.3	N/D	49.6	42.4	4.5	1.2	0.5	0.5	N/D	N/D	This study
	KOG06	N/D	5.4	N/D	1.4	N/D	21.7	64.6	4.6	1.3	1.0	N/D	N/D	N/D	This study
	KOG07	N/D	2.8	N/D	N/D	N/D	42.9	48.1	3.5	1.2	1.0	0.5	N/D	N/D	This study
	KOG08	N/D	5.2	N/D	N/D	N/D	12.3	58.4	22.1	0.6	1.0	0.4	N/D	N/D	This study
	KOG09	N/D	3.4	N/D	N/D	N/D	32.7	35.1	26.5	1.2	0.7	0.4	N/D	N/D	This study
	KOG10	N/D	4.1	N/D	1.0	N/D	23.3	40.0	29.5	1.2	0.6	0.3	N/D	N/D	This study
	Average	N/D	3.8	0.01	0.5	0.1	29.9	44.9	18.7	1.0	0.8	0.4	N/D	0.03	This study
Urumqi Glacier No.1	Average	N/A	N/A	N/A	N/A	N/A	60.2	3.7	16.1	0.6	N/D	N/D	N/A	N/D	9
Qiyi Glacier	Average	0.8	15.4	0.01	3.5	1.6	52.2	7.6	14.4	1.3	2.8	0.3	0.2	N/D	13
Dongkemadi Glacier	Average	0.4	3.9	0.04	3.1	1.8	74.3	4.7	5.3	5.1	N/D	N/D	1.3	N/D	15
Hailuogou Glacier	Average	N/A	N/A	N/A	N/A	N/A	30.6	11.3	16.6	0.9	N/D	N/D	N/A	6.2	17

Note. N/D denotes not been detected in the laboratory, and N/A denotes no available data.

Supplementary Table 8: Cation fluxes (±std; Gg year^{-1}) and crustal cation denudation rates (CDR; ±std; Σ^{*}meq^{+} m^{-2} year^{-1}) for glaciers worldwide calculated by the multiplication of regional discharge-weighted mean cation concentrations (μeq L^{-1}) by total glacial runoff globally (RUN; km^{3} year^{-1}) based on all data (G1) from 63 non-ice sheet glaciers in nine glacial regions (ASG, ALG, ACG, CEG, ICG, LLG, SCG, SJG, and CUG; Supplementary Table 3), in comparison to those calculated based on the mid-summer (July-August) data (G2) from 24 non-ice sheet glaciers in six glacial regions (ASG, CEG, ICG, LLG, SCG, and CUG; Supplementary Table 9) in the current global dataset. N denotes sample size.

	RUN [c]	Regional discharge-weighted mean concentration				Cation flux					
		Na^{+}	K^{+}	Mg^{2+}	Ca^{2+}	Na^{+}	K^{+}	Mg^{2+}	Ca^{2+}	Total	N
G1 [a]	1430	97.7±96.2	28.2±19.8	198±182	834±246	3212±3164	1573±1104	3391±3131	23,859±7047	32,035±14,446	>4641
G2 [b]	1430	62.5±81.8	19.1±25.1	207±177	485±349	2056±2691	1063±1398	3552±3043	13,876±9984	20,546±17,117	>593

Note. All cation concentrations were not corrected for sea-salt contribution, and N/A denotes no available data.

[a] Regional discharge-weighted mean concentrations were calculated by regional runoff multiplied by regional mean Na^{+}, K^{+}, Mg^{2+} and Ca^{2+} concentrations from nine non-ice sheet glacial regions (Supplementary Table 5) and then the sum of their product was divided by glacial runoff from these nine glacial regions (1130 km^{3} year^{-1}; Table 1).

[b] Regional discharge-weighted mean concentrations were calculated by regional runoff multiplied by regional mean Na^{+}, K^{+}, Mg^{2+} and Ca^{2+} concentrations from six non-ice sheet glacial regions (Supplementary Table 9) and then the sum of their product was divided by glacial runoff from these six glacial regions (507 km^{3} year^{-1}; Table 1).

[c] Value from Bliss and others4.

Supplementary Table 9: Mean cation concentrations (Con; ±std; μeq L^{-1}) for 24 non-ice sheet glaciers sampled during the mid-summer period (July-August in the northern Hemisphere) in six glacial regions (ASG, CEG, ICG, LLG, SCG, and CUG) worldwide in the current global dataset (Supplementary Table 3). N denotes sample size.

Region	Glacier	Sampling period	Na^+		K^+		Mg^{2+}		Ca^{2+}		
			Con	Std	Con	Std	Con	Std	Con	Std	N
ASG	Levyi Aktru Glacier	Jul, 2012	20.0	N/A	20.5	N/A	153	N/A	750	N/A	1
	Bogeda Glacier	Jul, 2008	21.4	N/A	2.79	N/A	45.4	N/A	228	N/A	72
	Heigou Glacier	Aug, 2008	26.6	N/A	2.31	N/A	41.9	N/A	211	N/A	50
	Koxkar Glacier	Jul, 2008	87.4	N/A	47.6	N/A	71.4	N/A	365	N/A	31
	Qingbingtan Glacier	Aug, 2008	359	N/A	131	N/A	527	N/A	889	N/A	30
	Shuiguanhe Glacier No.2	Jul, 2007	49.2	N/A	13.9	N/A	549	N/A	468	N/A	24
	Shuiguanhe Glacier No.3	Jul, 2007	23.2	N/A	10.7	N/A	512	N/A	436	N/A	24
	Shuiguanhe Glacier No.4	Jul, 2007	19.4	N/A	6.44	N/A	365	N/A	242	N/A	24
	Yanglonghe Glacier No.1	Aug, 2007	18.3	N/A	12.6	N/A	215	N/A	754	N/A	25
	Yanglonghe Glacier No.5	Aug, 2007	24.7	N/A	13.1	N/A	280	N/A	698	N/A	10
	Laohugou Glacier	Aug, 2007	42.8	N/A	17.8	N/A	231	N/A	487	N/A	24
	Qiyi Glacier	Aug, 2010	98.3	N/A	14.3	N/A	682	N/A	1545	N/A	12
	Kartamak Glacier	Aug, 2003	8.19	N/A	8.74	N/A	14.5	N/A	311	N/A	13
	Chhota Shigri Glacier	Jul-Aug, 1987	30.9	N/A	25.1	N/A	11.7	N/A	117	N/A	31
	Sutri Dhaka Glacier	Jul-Aug, 2015	5.00	N/A	14.7	N/A	43.6	N/A	242	N/A	59
	Average	N/A	55.6	88.1	22.7	31.7	249	226	516	369	430
CEG	Gornergletscher	Jul-Aug, 1974	19.1	N/A	13.1	N/A	77.5	N/A	216	N/A	69
		Jul-Aug, 1976	13.5	N/A	15.1	N/A	88.3	N/A	253	N/A	41
	Marmolada Glacier	Aug, 1990	N/A	N/A	N/A	N/A	46.4	N/A	713	N/A	6
	Average	N/A	16.3	4.00	14.1	1.45	70.8	21.8	394	277	116
ICG	Fjallsjökull Glacier	Jul/Aug, 1976	83.7	N/A	5.53	N/A	51.2	N/A	243	N/A	18
	Vatnajökull Glacier	Jul, 2011	278	N/A	9.64	N/A	176	N/A	892	N/A	1
	Average	N/A	181	137	7.59	2.90	114	88.3	568	459	19
LLG	Rio Quilcay	Jul, 2008	47.6	N/A	14.9	N/A	495	N/A	1017	N/A	16
	Average	N/A	47.6	N/A	14.9	N/A	495	N/A	1017	N/A	16
SCG	Austre Okstindbreen	Jul, 1983	54.8	N/A	7.95	N/A	20.8	N/A	17.5	N/A	N/A
		Jul, 1984	29.1	N/A	5.90	N/A	8.33	N/A	11.5	N/A	N/A
		Jul, 1985	23.0	N/A	14.9	N/A	27.5	N/A	165	N/A	N/A

		Jul, 1986	29.1	N/A	28.7	N/A	41.7	N/A	283	N/A	N/A
		Jul, 1987	34.8	N/A	5.90	N/A	15.0	N/A	28.0	N/A	N/A
		Jul, 1988	14.8	N/A	4.36	N/A	14.2	N/A	39.5	N/A	N/A
		Jul, 1989	137	N/A	8.97	N/A	40.8	N/A	33.5	N/A	N/A
		Jul, 1990	75.2	N/A	6.92	N/A	23.3	N/A	33.0	N/A	N/A
	Average	N/A	49.7	40.0	10.4	8.04	24.0	12.2	76.3	96.6	N/A
CUG	Berendon Glacier	Jul-Aug, 1975	4.35	N/A	2.77	N/A	10.4	N/A	428	N/A	2
	Eliot Glacier	Jul, 2005	39.0	N/A	5.30	N/A	42.0	N/A	86.0	N/A	10
	South Cascade Glacier	Aug, 1992	9.57	N/A	23.2	N/A	10.5	N/A	71.4	N/A	N/A
	Average	N/A	17.6	18.7	10.4	11.1	21.0	18.2	195	202	>12

Note. N/A denotes no available data.

Supplementary Table 10: Cation denudation rates (CDR; $\Sigma^{*}meq^{+}$ m^{-2} $year^{-1}$) at > 29 glacial basins in nine glacial regions in current global dataset, in comparison to the longitude (LON; °) and latitude (LAT; °), basin area (Area; km^{2}) and ice cover (Cover; %), mean annual air temperature (MAT; ℃), mean annual precipitation (MAP; mm) and specific discharge (SQ; m $year^{-1}$). These glaciers include 6 glaciers in Asia (ASG), 1 glacier in Alaska (ALG), > 1 glacier in Arctic Canada (ACG), 3 glaciers in Central Europe (CEG), 3 glaciers in Iceland (ICG), 9 glaciers in Svalbard and Jan Mayen (SJG), 2 glaciers in Western Canada and USA (CUG), > 3 outlet glaciers in Greenland Periphery (GPG), and 1 outlet glacier in Antarctic and Subantarctic (ANG).

Region	Glacier	LON	LAT	Geology	Area (Cover)	MAT [a]	MAP [a]	SQ	CDR	Source
ASG	Batura Glacier	74.70	37.30	Carbonate-rich	760 (48)	-10.5	750	1.60	1600	24
	Chhota Shigri Glacier	77.52	32.23	Plutonic/metamorphic	40 (25)	6.03	1709	3.50	750	24,27,28,69
	Dokriani Glacier	78.43	33.57	Plutonic/metamorphic	9.58 (60)	-12.0	328	1.12	4160	31,70
	Dongkemadi Glacier	92.07	33.07	Carbonate-rich	28.0 (57)	-6.94	676	0.98	180 [b]	15,71
	Urumqi Glacier No.1	86.82	43.10	Carbonate-rich	3.34 (49)	-4.30	515	0.88	976	9
	Hailuogou Glacier	102.0	29.57	Carbonate-rich	80.5 (45)	-1.24	1353	4.63	2850	17
		102.0	29.57	Carbonate-rich	80.5 (45)	-1.24	1353	4.37	3108	17
ALG	Worthington Glacier	-145.7	61.17	Plutonic/metamorphic	13.0 (83)	-3.75	2390	7.70	1600	69
ACG	Lewis River	-74.00	69.00	Plutonic/metamorphic	205 (89)	-11.8	397	0.71	94.0	69,72
CEG	Haut Glacier d'Arolla	8.38	46.45	Plutonic/metamorphic	11.7 (54)	5.13	1778	1.70	640	73
		8.38	46.45	Plutonic/metamorphic	11.7 (54)	5.13	1778	2.30	685	73
	Gornergletscher	8.00	47.00	Plutonic/metamorphic	82.0 (83)	1.12	1844	1.34	478	74
	Glacier de Tsidjiore Nouve	8.25	46.95	Plutonic/metamorphic	4.80 (67)	-0.52	1765	1.20	510	75,76
ICG	Tungufljót Glacier	-20.42	64.43	Basalt-rich	720 (35)	4.50	1655	2.10	719	77
	Hvítá-S Glacier	-20.51	64.67	Basalt-rich	2000 (19)	-0.25	1689	2.10	1082	77
	Hvítá-W Glacier	-19.65	64.49	Basalt-rich	1685 (21)	0.76	2202	1.80	650	77
SJG	Erikbreen	12.39	79.61	Carbonate-rich	12.4 (73)	-9.94	672	0.51	320	78
	Austre Broggerbreen	11.82	78.87	Sedimentary mix	9.90 (71)	-4.18	690	0.84	240	78
		11.82	78.87	Sedimentary mix	9.90 (69)	-4.18	690	1.30	270	78
	Hannabreen	15.48	77.03	Carbonate-rich	13.3 (70)	-6.25	715	0.80	320	78
	Erdmannbreen	14.07	77.82	Sedimentary mix	16.0 (80)	-6.24	554	0.81	190	78
	Midre Lovénbreen	11.50	78.94	Plutonic/metamorphic	5.00 (80)	-4.47	656	1.50	450	78
		11.50	78.94	Plutonic/metamorphic	7.40 (26)	-4.47	656	1.30	560	78

	Scott Turnerbreen	15.95	78.10	Sedimentary mix	12.8 (65)	-8.18	653	0.52	160	55
	Finsterwalderbreen	15.30	77.47	Sedimentary mix	68.0 (65)	-6.25	715	0.84	440	79
		15.30	77.47	Sedimentary mix	68.0 (43)	-6.25	715	0.35	210	79
	Longyearbreen	15.50	78.18	N/A	10.7 (N/A)	-7.83	656	0.34	322	53
	Rieperbreen	16.20	78.13	Shale-rich	N/A (34)	-8.18	653	0.40	292	75
CUG	South Cascade Glacier	-120.4	48.20	Plutonic/metamorphic	6.14 (62)	4.04	1507	3.90	676	59,80
	Berendon Glacier	-129.5	53.48	Plutonic/metamorphic	53.0 (40)	7.41	3460	3.70	947	57
GPG	Kuannersuit Glacier	-53.28	69.67	Basalt-rich	258 (94)	-5.97	584	2.50	853	81
	Watson River	-50.00	67.00	N/A	9743 (94)	-9.55	411	0.38	38.0	82
		-50.00	67.00	N/A	9743 (94)	-9.55	411	0.29	40.0	82
		-50.00	67.00	N/A	9743 (94)	-9.55	411	0.26	36.0	82
		-50.00	67.00	N/A	9743 (94)	-9.55	411	0.56	56.0	82
	Mittivakkat Glacier	-37.84	65.69	Plutonic/metamorphic	N/A (N/A)	-2.90	1272	3.27	270	Unpublished
ANG	Tuva Glacier	-45.50	60.70	Plutonic/metamorphic	1.01 (N/A)	-1.56	1079	0.53	163	75

Note. N/A denotes data not available.

[a] Value was derived from ERA-Interim (ERA-I) datasets and was calculated by the method (see Table 1 in the main text).

[b] Value was corrected by the mean contribution of crustal-derived cation fluxes to total cation fluxes from Batura Glacier and Hailuogou Glacier on the Tibetan Plateau (79.9 % for Na^+, 99.7 % for K^+, 98.6 % for Mg^{2+}, and 99.9 % for Ca^{2+})24,83.

Supplementary References:

1 Reimann, C. & de Caritat, P. *Chemical Elements in the Environment-factsheets for the Geochemist and Environmental Scientist*. (Springer, 1998).

2 Langmuir, D. *Aqueous Environmental Geochemistry*. 600 (Prentice-Hall, New Jersey, 1997).

3 Pfeffer, W. T. *et al.* The Randolph Glacier Inventory: a globally complete inventory of glaciers. *Journal of Glaciology* **60**, 537-552, doi:10.3189/2014JoG13J176 (2014).

4 Bliss, A., Hock, R. & Radić, V. Global response of glacier runoff to twenty-first century climate change. *Journal of Geophysical Research: Earth Surface* **119**, 717-730, doi:10.1002/2013jf002931 (2014).

5 Savichev, O. G. & Paromov, V. V. Chemical composition of glacial meltwaters and river waters within the Aktru River basin (Gornyi Altai). *Geography and Natural Resources* **34**, 364-370, doi:10.1134/s1875372813040100 (2013).

6 Li, C., Hou, S. & Qin, D. Spatial differences of hydro-chemical and its control factors of the headwater runoff in the Urumqi River, Tianshan Mountains. *Journal of Glaciology and Geocryology* **25**, 72-76, doi:CNKI:SUN:BCDT.0.2003-01-010 (2003).

7 Liu, F., Williams, M. & Sun, J. Hydrochemical process and hydrological separation at the headwaters of the Urumqi River, Tianshan Mountains, China. *Journal of Glaciology and Geocryology* **21**, 362-370 (1999).

8 Feng, F., Li, Z., Jin, S., Dong, Z. & Wang, F. Hydrochemical characteristics and solute dynamics of meltwater runoff of Urumqi Glacier No.1, eastern Tianshan, northwest China. *Journal of Mountain Science* **9**, 472-482, doi:10.1007/s11629-012-2316-7 (2012).

9 Li, X. *et al.* Seasonal controls of meltwater runoff chemistry and chemical weathering at Urumqi Glacier No.1 in central Asia. *Hydrological Processes* **33**, 3258-3281, doi:10.1002/hyp.13555 (2019).

10 Wang, J., Ding, Y., Xu, J. & Han, H. Hydrochemical characteristic analysis of meltwater flow in Koxkar Glacier, Tianshan (west) Mountains. *Environmental Sciences* **27**, 1305-1311 (2006).

11 Zhao, A., Zhang, M., Li, Z., Wang, F. & Wang, S. Hydrochemical characteristics at the Glacier No.72 of Qingbingtan, Tomur Peak. *Environmental Sciences* **33**, 1484-1490 (2012).

12 Wu, X., Li, Q., Song, G., He, J. & Jiang, X. Hydrochemical characteristics and evolution of runoff at Qiyi Glacier, Qilian Mountains. *Environmental Sciences* **29**, 613-618, doi:10.3321/j.issn:0250-3301.2008.03.012 (2008).

13 Li, X., Qin, D., Jing, Z., Li, Y. & Wang, N. Diurnal hydrological controls and non-filtration effects on minor and trace elements in stream water draining the Qiyi Glacier, Qilian Mountain. *Science China Earth Sciences* **56**, 81-92, doi:10.1007/s11430-012-4480-6 (2013).

14 Wu, X. Diurnal and seasonal variation of glacier meltwater hydrochemistry in Qiyi glacierized catchment in Qilian Mountains, northwest China: Implication for chemical weathering. *Journal of Mountain Science* **15**, 1035-1045, doi:10.1007/s11629-017-4695-2 (2018).

15 Li, X. *et al.* Diurnal dynamics of minor and trace elements in stream water draining Dongkemadi Glacier on the Tibetan Plateau and its environmental implications. *Journal of Hydrology* **541**, 1104-1118, doi:10.1016/j.jhydrol.2016.08.021 (2016).

16 Zhao, H., Yao, T. & Xu, B. Hydrological and hydrochemical features of Kartamak Glacier area in Muztag Ata. *Journal of Glaciology and Geocryology* **28**, 269-275 (2006).

17 Li, X. *et al.* Intense chemical weathering at glacial meltwater-dominated Hailuogou basin in the southeastern Tibetan Plateau. *Water* **11**, 1209, doi:10.3390/w11061209 (2019).

18 Liu, L., Ren, J. & Qin, D. Chemical characteristics at the head of Rongbuk River on Mt. Everest. *Environmental Sciences* **21**, 59–63 (2000).

19 Zhang, F. *et al.* Meltwater hydrochemistry at four glacial catchments in the headwater of Indus River. *Environmental Science and Pollution Research* **26**, 23645-23660, doi:10.1007/s11356-019-05422-5 (2019).

20 Singh, V. B., Ramanathan, A. L. & Kuriakose, T. Hydrogeochemical assessment of meltwater quality using major ion chemistry: a case study of Bara Shigri Glacier, western Himalaya, India. *National Academy Science Letters* **38**, 147-151, doi:10.1007/s40009-014-0310-z (2014).

21 Singh, V. B. & Ramanathan, A. L. Assessment of solute and suspended sediments acquisition processes in the Bara Shigri Glacier meltwater (Western Himalaya, India). *Environmental Earth Sciences* **74**, 2009-2018, doi:10.1007/s12665-015-4584-3 (2015).

22 Singh, V. B. & Ramanathan, A. L. Characterization of hydrogeochemical processes controlling major ion chemistry of the Batal Glacier meltwater, Chandra basin, Himachal Pradesh, India. *Proceedings of the National Academy of Sciences, India Section A: Physical Sciences* **87**, 145-153, doi:10.1007/s40010-016-0294-9 (2016).

23 Singh, V. B., Keshari, A. K. & Ramanathan, A. L. Major ion chemistry and atmospheric CO_2 consumption deduced from the Batal Glacier, Lahaul–Spiti valley, Western Himalaya, India. *Environment, Development and Sustainability* **22**, 6585-6603, doi:10.1007/s10668-019-00501-6 (2019).
24 Hodson, A., Porter, P., Lowe, A. & Mumford, P. Chemical denudation and silicate weathering in Himalayan glacier basins: Batura Glacier, Pakistan. *Journal of Hydrology* **262**, 193-208, doi:org/10.1016/S0022-1694(02)00036-7 (2002).
25 Kumar, R. *et al.* Hydro-geochemical analysis of meltwater draining from Bilare Banga Glacier, Western Himalaya. *Acta Geophysica*, doi:10.1007/s11600-019-00262-w (2019).
26 Singh, V. B., Ramanathan, A. L., Pottakkal, J. G. & Kumar, M. Hydrogeochemistry of meltwater of the Chaturangi Glacier, Garhwal Himalaya, India. *Proceedings of the National Academy of Sciences, India Section A: Physical Sciences*, doi:10.1007/s40010-014-0181-1 (2014).
27 Hasnain, S. I., Subramanian, V. & Dhanpal, K. Chemical characteristics and suspended sediment load of meltwaters from a Himalayan glacier in India. *Journal of Hydrology* **106**, 99-108, doi:https://doi.org/10.1016/0022-1694(89)90168-6 (1989).
28 Sharma, P., Ramanathan, A. L. & Pottakkal, J. Study of solute sources and evolution of hydrogeochemical processes of the Chhota Shigri Glacier meltwaters, Himachal Himalaya, India. *Hydrological Sciences Journal* **58**, 1128-1143, doi:10.1080/02626667.2013.802092 (2013).
29 Singh, V. B., Ramanathan, A. L., Sharma, P. & Pottakkal, J. G. Dissolved ion chemistry and suspended sediment characteristics of meltwater draining from Chhota Shigri Glacier, western Himalaya, India. *Arabian Journal of Geosciences* **8**, 281-293, doi:10.1007/s12517-013-1176-y (2015).
30 Singh, V. B. & Ramanathan, A. L. Hydrogeochemistry of the Chhota Shigri Glacier meltwater, Chandra basin, Himachal Pradesh, India: solute acquisition processes, dissolved load and chemical weathering rates. *Environmental Earth Sciences* **76**, doi:10.1007/s12665-017-6465-4 (2017).
31 Hasnain, S. I. & Thayyen, R. Controls on the major-ion chemistry of the Dokriani Glacier meltwaters, Ganga basin, Garhwal Himalaya, India. *Journal of Glaciology* **45**, 87-92, doi:10.1017/S0022143000003063 (1999).
32 Ahmad, S. & Hasnain, S. I. Chemical characteristics of stream draining from Dudu Glacier: an alpine meltwater stream in Ganga headwater, Garhwal Himalaya. *Journal of China University of Geosciences* **12**, 75-83 (2001).
33 Kumar, K. *et al.* Solute dynamics of meltwater of Gangotri Glacier, Garhwal Himalaya, India. *Environmental Geology* **58**, 1151-1159, doi:10.1007/s00254-008-1592-6 (2009).
34 Singh, V. B. *et al.* Chemical characterisation of meltwater draining from Gangotri Glacier, Garhwal Himalaya, India. *Journal of Earth System Science* **121**, 625-636 (2012).
35 Singh, V. B., Ramanathan, A. L., Pottakkal, J. G. & Kumar, M. Seasonal variation of the solute and suspended sediment load in Gangotri Glacier meltwater, central Himalaya, India. *Journal of Asian Earth Sciences* **79**, 224-234, doi:10.1016/j.jseaes.2013.09.010 (2014).
36 Sharma, M. K., Thayyen, R. J., Jain, C. K., Arora, M. & Lal, S. Assessment of system characteristics of Gangotri Glacier headwater stream. *Science of the Total Environment* **662**, 842-851, doi:10.1016/j.scitotenv.2019.01.229 (2019).
37 Tuladhar, A., Kayastha, R. B., Gurung, S. & Shrestha, A. Hydro-chemical characterization of glacial melt waters draining from Langtang Valley, Nepal. *Journal of Water Resource and Protection* **07**, 605-613, doi:10.4236/jwarp.2015.78049 (2015).
38 Singh, V. B. *et al.* Evaluation of meltwater quality using dissolved ions chemistry and multivariate statistical methods: a case study of the Manimahesh Glacier, Ravi basin, Himachal Pradesh, India. *Proceedings of the National Academy of Sciences, India Section A: Physical Sciences* **90**, 57-66, doi:10.1007/s40010-018-0560-0 (2018).
39 Singh, V. B., Ramanathan, A. L. & Sharma, P. Major ion chemistry and assessment of weathering processes of the Patsio Glacier meltwater, Western Himalaya, India. *Environmental Earth Sciences* **73**, 387-397, doi:10.1007/s12665-014-3432-1 (2015).
40 Singh, A. T., Laluraj, C. M., Sharma, P., Patel, L. K. & Thamban, M. Export fluxes of geochemical solutes in the meltwater stream of Sutri Dhaka Glacier, Chandra basin, Western Himalaya. *Environmental Monitoring and Assessment* **189**, 555, doi:10.1007/s10661-017-6268-9 (2017).
41 Kumar, R. *et al.* Hydro-geochemical characteristics of glacial meltwater from Naradu Glacier catchment, Western Himalaya. *Environmental Earth Sciences* **78**,

doi:10.1007/s12665-019-8687-0 (2019).
42 Anderson, S. P., Drever, J. I., Frost, C. D. & Holden, P. Chemical weathering in the foreland of a retreating glacier. *Geochimica et Cosmochimica Acta* **64**, 1173-1189 (2000).
43 Anderson, S. P., Longacre, S. A. & Kraal, E. R. Patterns of water chemistry and discharge in the glacier-fed Kennicott River, Alaska: evidence for subglacial water storage cycles. *Chemical Geology* **202**, 297-312, doi:10.1016/j.chemgeo.2003.01.001 (2003).
44 Skidmore, M. L. & Sharp, M. J. Drainage system behaviour of a High-Arctic polythermal glacier. *Annals of Glaciology* **28**, 209-215 (1999).
45 Hindshaw, R. S. *et al.* Hydrological control of stream water chemistry in a glacial catchment (Damma Glacier, Switzerland). *Chemical Geology* **285**, 215-230, doi:10.1016/j.chemgeo.2011.04.012 (2011).
46 Fairchild, I. J., Bradby, L., Sharp, M. & Tison, J.-L. Hydrochemistry of carbonate terrains in alpine glacial settiings. *Earth Surface Processes and Landforms* **19**, 33-54 (1994).
47 Collins, D. N. Hydrochemistry of meltwaters draining from an alpine glacier. *Arctic and Alpine Research* **11**, 307-324 (1979).
48 Mitchell, A. C., Brown, G. H. & Fuge, R. Minor and trace element export from a glacierized Alpine headwater catchment (Haut Glacier d'Arolla, Switzerland). *Hydrological Processes* **15**, 3499-3524, doi:10.1002/hyp.1041 (2001).
49 Raiswell, R. & Thomas, A. G. Solute acquisition in glacial melt waters. I. Fjallsjökull (South-East Iceland): Bulk melt waters with closed-system characteristics. *Journal of Glaciology* **30**, 35-43 (1984).
50 Galeczka, I., Oelkers, E. H. & Gislason, S. R. The chemistry and element fluxes of the July 2011 Múlakvísl and Kaldakvísl glacial floods, Iceland. *Journal of Volcanology and Geothermal Research* **273**, 41-57, doi:10.1016/j.jvolgeores.2013.12.004 (2014).
51 Fortner, S. K. *et al.* Elevated stream trace and minor element concentrations in the foreland of receding tropical glaciers. *Applied Geochemistry* **26**, 1792-1801, doi:10.1016/j.apgeochem.2011.06.003 (2011).
52 Theakstone, W. H. & Knudsen, N. T. Isotopic and ionic variations in glacier river water during three contrasting ablation seasons. *Hydrological Processes* **10**, 523-539 (1996).
53 Yde, J. C., Riger-Kusk, M., Christiansen, H., Knudsen, N. & Humlum, O. Hydrochemical characteristics of bulk meltwater from an entire ablation season, Longyearbreen, Svalbard. *Journal of Glaciology* **54**, 259-272, doi:10.3189/002214308784886234 (2008).
54 Rutter, N., Hodson, A., Irvine-Fynn, T. & Kristensen, M. Hydrology and hydrochemistry of a deglaciating high-Arctic catchment, Svalbard. *Journal of Hydrology* **410**, 39-50, doi:10.1016/j.jhydrol.2011.09.001 (2011).
55 Hodgkins, R., Tranter, M. & Dowdeswell, J. A. Solute provenance, transport and denudation in a high arctic glacierized catchment. *Hydrological Processes* **11**, 1813-1832, doi:10.1002/(sici)1099-1085(199711)11:14<1813::Aid-hyp498>3.0.Co;2-c (1997).
56 Stachnik, Ł. *et al.* Chemical denudation and the role of sulfide oxidation at Werenskioldbreen, Svalbard. *Journal of Hydrology* **538**, 177-193, doi:10.1016/j.jhydrol.2016.03.059 (2016).
57 Eyles, N., Sasseville, D. R., Slatt, R. M. & Rogerson, R. J. Geochemical denudation rates and solute transport mechanisms. *Canadian Journal of Earth Sciences* **19**, 1570-1581, doi:doi.org/10.1139/e82-135 (1982).
58 Fortner, S. K., Lyons, W. B., Fountain, A. G., Welch, K. A. & Kehrwald, N. M. Trace element and major ion concentrations and dynamics in glacier snow and melt: Eliot Glacier, Oregon Cascades. *Hydrological Processes* **23**, 2987-2996, doi:10.1002/hyp.7418 (2009).
59 Axtmann, E. & Stallard, R. Chemical weathering in the South Cascade Glacier basin, comparison of subglacial and extra-glacial weathering. *Biogeochemistry of Seasonally Snow-covered Catchments* **228** (1995).
60 Aciego, S. M., Stevenson, E. I. & Arendt, C. A. Climate versus geological controls on glacial meltwater micronutrient production in southern Greenland. *Earth and Planetary Science Letters* **424**, 51-58, doi:10.1016/j.epsl.2015.05.017 (2015).
61 Graly, J., Harrington, J. & Humphrey, N. Combined diurnal variations of discharge and hydrochemistry of the Isunnguata Sermia outlet, Greenland Ice Sheet. *The Cryosphere* **11**, 1131-1140, doi:10.5194/tc-11-1131-2017 (2017).
62 Hindshaw, R. S., Rickli, J., Leuthold, J., Wadham, J. & Bourdon, B. Identifying weathering sources and processes in an outlet glacier of the Greenland Ice Sheet using Ca and Sr isotope ratios. *Geochimica et Cosmochimica Acta* **145**, 50-71, doi:10.1016/j.gca.2014.09.016 (2014).
63 Urra, A. *et al.* Weathering dynamics under contrasting Greenland ice sheet catchments. *Frontiers in Earth Science* **7**, 299, doi:10.3389/feart.2019.00299 (2019).

64 Wimpenny, J. *et al.* Glacial effects on weathering processes: New insights from the elemental and lithium isotopic composition of West Greenland rivers. *Earth and Planetary Science Letters* **290**, 427-437, doi:10.1016/j.epsl.2009.12.042 (2010).

65 Andrews, M. G., Jacobson, A. D., Osburn, M. R. & Flynn, T. M. Dissolved carbon dynamics in meltwaters from the Russell Glacier, Greenland Ice Sheet. *Journal of Geophysical Research: Biogeosciences* **123**, 2922-2940, doi:10.1029/2018jg004458 (2018).

66 Yde, J. C. & Knudsen, N. The importance of oxygen isotope provenance in relation to solute content of bulk meltwaters at Imersuaq Glacier, West Greenland. *Hydrological Processes* **18**, 125-139, doi:10.1002/hyp.1317 (2004).

67 Bhatia, M. P. *et al.* Organic carbon export from the Greenland ice sheet. *Geochimica et Cosmochimica Acta* **109**, 329-344, doi:10.1016/j.gca.2013.02.006 (2013).

68 Stumpf, A. R. *et al.* Glacier meltwater stream chemistry in Wright and Taylor Valleys, Antarctica: Significant roles of drift, dust and biological processes in chemical weathering in a polar climate. *Chemical Geology* **322-323**, 79-90, doi:10.1016/j.chemgeo.2012.06.009 (2012).

69 Anderson, S., Drever, J. & Humphrey, N. Chemical weathering in glacial environments. *Geology* **25**, 399-402, doi:10.1130/0091-7613(1997)025<0399:CWIGE>2.3.CO (1997).

70 Hasnain, S. I. & Thayyen, R. Sediment transport and solute variation in meltwaters of Dokriani glacier (Bamak), Garhwal Himalaya. *Journal of the Geological Society of India* **47**, 731-739 (1996).

71 Li, X. *et al.* Corrigendum to "Diurnal dynamics of minor and trace elements in stream water draining Dongkemadi Glacier on the Tibetan Plateau and its environmental implications" [J. Hydrol. 541 (2016) 1104–1118]. *Journal of Hydrology* **555**, doi:10.1016/j.jhydrol.2017.11.019 (2017).

72 Church, M. On the quality of some waters on Baffin Island, northwest territories. *Canadian Journal of Earth Sciences* **11**, 1676-1688, doi:10.1139/e74-166 (1974).

73 Sharp, M., Tranter, M., Brown, G. H. & Skidmore, M. Rates of chemical denudation and CO_2 drawdown in a glacier-covered alpine catchment. *Geology* **23**, 61-64, doi:10.1130/0091-7613(1995)023<0061:Rocdac>2.3.Co;2 (1995).

74 Collins, D. N. Solute yield from a glacierized high mountain basin (Gornera, Gornergletscher, Switzerland). *Dissolved loads of rivers and surface water quantity/quality relationships. Proc. Hamburg symposium, 1983*, 41-49 (1983).

75 Hodson, A., Heaton, T., Langford, H. & Newsham, K. Chemical weathering and solute export by meltwater in a maritime Antarctic glacier basin. *Biogeochemistry* **98**, 9-27, doi:10.1007/s10533-009-9372-2 (2010).

76 Souchez, R. A. & Lemmens, M. M. *Solutes. In Glacio-fluvial Sediment Transfer: An Alpine Perspective*. 285-303 (Wiley, 1987).

77 Gislason, S. R., Arnorsson, S. & Armannsson, H. Chemical weathering of basalt in Southwest Iceland: Effects of runoff, age of rocks and vegetative/glacial cover. *American Journal of Science* **296**, 837-907, doi:10.2475/ajs.296.8.837 (1996).

78 Hodson, A., Tranter, M. & Vatne, G. Contemporary rates of chemical denudation and atmospheric CO_2 sequestration in glacier basins: An Arctic perspective. *Earth Surface Processes and Landforms* **25**, 1447-1471, doi:10.1002/1096-9837(200012)25:13<1447::AID-ESP156>3.0.CO;2-9 (2000).

79 Wadham, J. L., Hodson, A. J., Tranter, M. & Dowdeswell, J. A. The rate of chemical weathering beneath a quiescent, surge-type, polythermal-based glacier, southern Spitsbergen, Svalbard. *Annals of Glaciology* **24**, 27-31, doi:10.3189/S0260305500011885 (1997).

80 Reynolds, R. C. & Johnson, N. M. Chemical weathering in the temperate glacial environment of the Northern Cascade Mountains. *Geochimica et Cosmochimica Acta* **36**, 537-554, doi:10.1016/0016-7037(72)90074-9 (1972).

81 Yde, J. C., Tvis Knudsen, N. & Nielsen, O. B. Glacier hydrochemistry, solute provenance, and chemical denudation at a surge-type glacier in Kuannersuit Kuussuat, Disko Island, West Greenland. *Journal of Hydrology* **300**, 172-187, doi:10.1016/j.jhydrol.2004.06.008 (2005).

82 Yde, J. C., Knudsen, N. T., Hasholt, B. & Mikkelsen, A. B. Meltwater chemistry and solute export from a Greenland Ice Sheet catchment, Watson River, West Greenland. *Journal of Hydrology* **519**, 2165-2179, doi:10.1016/j.jhydrol.2014.10.018 (2014).

83 Li, X. *Hydrochemical Characteristics of Meltwater from Glaciers at the Typical Catchments in Western China* Doctor thesis, University of Chinese Academy of Sciences, (2009).
